# Finite element approximation of the Laplace–Beltrami operator on a surface with boundary

**DOI:** 10.1007/s00211-018-0990-2

**Published:** 2018-07-14

**Authors:** Erik Burman, Peter Hansbo, Mats G. Larson, Karl Larsson, André Massing

**Affiliations:** 1 Department of Mathematics, University College London, London, WC1E 6BT UK; 2 Department of Mechanical Engineering, Jönköping University, 55111 Jönköping, Sweden; 3 Department of Mathematics and Mathematical Statistics, Umeå University, 90187 Umeå, Sweden

**Keywords:** 65M60, 65M85

## Abstract

We develop a finite element method for the Laplace–Beltrami operator on a surface with boundary and nonhomogeneous Dirichlet boundary conditions. The method is based on a triangulation of the surface and the boundary conditions are enforced weakly using Nitsche’s method. We prove optimal order a priori error estimates for piecewise continuous polynomials of order $k \ge 1$ in the energy and $L^2$ norms that take the approximation of the surface and the boundary into account.

## Introduction

Finite element methods for problems on surfaces have been rapidly developed starting with the seminal work of Dziuk [11]. Different approaches have been developed including methods based on meshed surfaces [1, 9, 10, 15, 17], and methods based on implicit or embedded approaches [5, 20, 21], see also the overview articles [3, 12], and the references therein. So far the theoretical developments are, however, restricted to surfaces without boundary.

In this contribution we develop a finite element method for the Laplace–Beltrami operator on a surface which has a boundary equipped with a nonhomogeneous Dirichlet boundary condition. The results may be readily extended to include Neumann conditions on part of the boundary, which we also comment on in a remark. The method is based on a triangulation of the surface together with a Nitsche formulation [19] for the Dirichlet boundary condition. Polynomials of order *k* are used both in the interpolation of the surface and in the finite element space. Our theoretical approach is related to the recent work [4] where a priori error estimates for a Nitsche method with so called boundary value correction [2] is developed for the Dirichlet problem on a (flat) domain in $\mathbb {R}^n$. Boundary value correction consists of using a modified bilinear form that compensates for the approximation of the boundary in such a way that higher order convergence may be obtained using for instance only piecewise linear approximation of the boundary. We also mention the work [23] where the smooth curved boundary of a domain in $\mathbb {R}^2$ is interpolated and Dirichlet boundary conditions are strongly enforced in the nodes.

Provided the error in the position of the approximate surface and its boundary is (pointwise) of order $k+1$ and the error in the normals/tangents is of order *k*, we prove optimal order error estimates in the $L^2$ and energy norms. No additional regularity of the exact solution, compared to standard estimates, is required. The proof is based on a Strang Lemma which accounts for the error caused by approximation of the solution, the surface, and the boundary. Here the discrete surface is mapped using a closest point mapping onto a surface containing the exact surface. The error caused by the boundary approximation is then handled using a consistency argument. Special care is required to obtain optimal order $L^2$ error estimates and a refined Aubin–Nitsche duality argument is used which exploits the fact that the solution to dual problem is small close to the boundary since the dual problem is equipped with a homogeneous Dirichlet condition. Even though our main focus in this contribution is the weak Nitsche method to handle the Dirichlet condition a standard strong implementation is also of interest and we therefore include a detailed description how strong boundary conditions may be implemented and analysed in our framework.

The outline of the paper is as follows: In Sect. 2 we formulate the model problem and finite element method. We also formulate the precise assumptions on the approximation of the surface and its boundary. In Sect. 3 we develop the necessary results to prove our main error estimates. In Sect. 4 we present numerical results confirming our theoretical findings.

## Model problem and method

### The surface

Let, $\Gamma \subset \widetilde{\Gamma }$ be a surface with smooth boundary $\partial \Gamma$, where $\widetilde{\Gamma }$ is a smooth closed connected hypersurface embedded in $\mathbb {R}^3$. We let *n* be the exterior unit normal to $\widetilde{\Gamma }$ and $\nu$ be the exterior unit conormal to $\partial \Gamma$, i.e. $\nu (x)$ is orthogonal both to the tangent vector of $\partial \Gamma$ at *x* and the normal *n*(*x*) of $\widetilde{\Gamma }$. For $\widetilde{\Gamma }$, we denote its associated signed distance function by $\rho$ which satisfies $\nabla \rho = n$, and we define an open tubular neighborhood of $\widetilde{\Gamma }$ by $U_{\delta } (\widetilde{\Gamma }) = \{ x \in \mathbb {R}^3 {:}\; |\rho (x)|< \delta \}$ with $\delta >0$. Then there is $\delta _{0,\widetilde{\Gamma }}>0$ such that the closest point mapping $p{:}\;U_{\delta _{0,\widetilde{\Gamma }}}(\widetilde{\Gamma }) \rightarrow \widetilde{\Gamma }$ assigns precisely one point on $\widetilde{\Gamma }$ to each point in $U_{\delta _{0,\widetilde{\Gamma }}}(\widetilde{\Gamma })$. The closest point mapping takes the form

2.1 $$\begin{aligned} p{:}\;U_{\delta _{0,\widetilde{\Gamma }}}(\widetilde{\Gamma }) \ni x \mapsto x - \rho (x) n \circ p(x) \in \widetilde{\Gamma }\end{aligned}$$

For the boundary curve $\partial \Gamma$, let $\rho _{\partial \Gamma }$ be the distance function to the curve $\partial \Gamma$, and $p_{\partial \Gamma }$ be the associated closest point mapping with associated tubular neighborhood $U_{\delta }(\partial \Gamma ) = \{x\in \mathbb {R}^3 {:}\; |\rho _{\partial \Gamma }(x)| < \delta \}$. Note that there is $\delta _{0,\partial \Gamma }>0$ such that the closest point mapping $p_{\partial \Gamma }{:}\;U_{\delta _{0,\partial \Gamma }}(\partial \Gamma ) \rightarrow \partial \Gamma$ is well defined. Finally, we let $\delta _0 = \min (\delta _{0,\widetilde{\Gamma }},\delta _{0,\partial \Gamma })$ and introduce $U_{\delta _0}(\Gamma ) = \{x\in \mathbb {R}^3 {:}\; |\rho (x)|\lesssim \delta _0\}$.

#### Remark 2.1

Clearly we may take $\widetilde{\Gamma }$ to be a surface that is only slightly larger than $\Gamma$ but for simplicity we have taken $\widetilde{\Gamma }$ closed in order to obtain a well defined closest point mapping without boundary effects in a convenient way.

### The problem

**Tangential calculus** For each $x \in \widetilde{\Gamma }$ let $T_x(\widetilde{\Gamma }) = \{y\in \mathbb {R}^3 {:}\; (y,n(x))_{\mathbb {R}^3} = 0\}$ and $N_x(\Gamma ) = \{y\in \mathbb {R}^3 {:}\; \alpha n(x), \alpha \in \mathbb {R}\}$ be the tangent and normal spaces equipped with the inner products $(v,w)_{T_x(\widetilde{\Gamma })} = (v,w)_{\mathbb {R}^3}$ and $(v,w)_{N_x(\widetilde{\Gamma })} = (v,w)_{\mathbb {R}^3}$. Let $P_\Gamma {:}\;\mathbb {R}^3 \rightarrow T_x(\widetilde{\Gamma })$ be the projection of $\mathbb {R}^3$ onto the tangent space given by $P_\Gamma = I - n \otimes n$ and let $Q_\Gamma {:}\;\mathbb {R}^3 \rightarrow N_x(\widetilde{\Gamma })$ be the orthogonal projection onto the normal space given by $Q_\Gamma = I - P_\Gamma = n \otimes n$. The tangent gradient is defined by $\nabla _\Gamma v = P_\Gamma \nabla v$. For a tangential vector field *w*, i.e. a mapping $w{:}\;\widetilde{\Gamma }\ni x \mapsto w(x) \in T_x(\widetilde{\Gamma })$, the divergence is defined by $\text {div}_\Gamma w = \text {tr}(w\otimes \nabla _\Gamma )$. Then the Laplace–Beltrami operator is given by $\Delta _\Gamma v = \text {div}_\Gamma \nabla _\Gamma v$. Note that we have Green’s formula

2.2 $$\begin{aligned} (-\Delta _\Gamma v,w)_{\Gamma } = (\nabla _\Gamma v, \nabla _\Gamma w)_{\Gamma } - (\nu \cdot \nabla _\Gamma v, w)_{\partial \Gamma } \end{aligned}$$

where $(\cdot ,\cdot )_\omega$ denotes the usual $L^2$ inner product on $\omega \subset \widetilde{\Gamma }$.

**Model problem** Find $u{:}\;\Gamma \rightarrow \mathbb {R}$ such that

2.3 $$\begin{aligned} -\Delta _\Gamma u= & {} f \quad \text {in }\Gamma \end{aligned}$$

2.4 $$\begin{aligned} u= & {} g \quad \text {on }\partial \Gamma \end{aligned}$$

where $f\in H^{-1}(\Gamma )$ and $g\in H^{1/2}(\partial \Gamma )$ are given data. Thanks to the Lax–Milgram theorem, there is a unique solution $u\in H^1(\Gamma )$ to this problem. Moreover, we have the elliptic regularity estimate

2.5 $$\begin{aligned} \Vert u\Vert _{H^{s+2}(\Gamma )} \lesssim \Vert f\Vert _{H^s(\Gamma )} + \Vert g\Vert _{H^{s+3/2}(\Gamma )}, \quad s \ge -1 \end{aligned}$$

since $\Gamma$ and $\partial \Gamma$ are smooth. Here and below we use the notation $\lesssim$ to denote less or equal up to a constant. We also adopt the standard notation $H^s(\omega )$ for the Sobolev space of order *s* on $\omega \subset \widetilde{\Gamma }$ with norm $\Vert \cdot \Vert _{H^s(\omega )}$. For $s=0$ we use the notation $L^2(\omega )$ with norm $\Vert \cdot \Vert _\omega$, see [24] for a detailed description of Sobolev spaces on smooth manifolds with boundary.

### The discrete surface and finite element spaces

To formulate our finite element method for the boundary value problem (2.3)–(2.4) in the next section, we here summarize our assumptions on the approximation properties of the discretization of $\Gamma$.

**Discrete surface** Let $\{\Gamma _h,\, h \in (0,h_0]\}$ be a family of connected triangular surfaces with, mesh parameter *h*, that approximates $\Gamma$ and let $\mathcal {K}_h$ be the mesh associated with ${\Gamma _h}$. For each element $K\in \mathcal {K}_h$, there is a bijection $F_K{:}\; \widehat{K} \rightarrow K$ such that $F_K \in [\widehat{V}_k]^3 = [P_k(\widehat{K})]^3$, where $\widehat{K}$ is a reference triangle in $\mathbb {R}^2$ and $P_k(\widehat{K})$ is the space of polynomials of order less or equal to *k*. We assume that the mesh is quasi-uniform. For each $K \in \mathcal {K}_h$, we let $n_h\vert _K$ be the unit normal to $\Gamma _h$, oriented such that $(n_h, n\circ p)_{\mathbb {R}^3}>0$. On the element edges forming $\partial \Gamma _h$, we define $\nu _{\partial \Gamma _h}$ to be the exterior unit conormal to $\partial \Gamma _h$, i.e. $\nu _{\partial \Gamma _h}(x)$ is orthogonal both to the tangent vector of $\partial \Gamma _h$ at *x* and the normal $n_h(x)$ of $\Gamma _h$. We also introduce the tangent projection $P_{{\Gamma _h}}= I - n_h \otimes n_h$ and the normal projection $Q_{{\Gamma _h}}= n_h \otimes n_h$, associated with ${\Gamma _h}$.

**Geometric approximation property** We assume that $\{\Gamma _h, h \in (0,h_0]\}$ approximate $\Gamma$ in the following way: for all $h\in (0,h_0]$ it holds

2.6 $$\begin{aligned}&\Gamma _h \subset U_{\delta _0}(\Gamma ) \end{aligned}$$

2.7 $$\begin{aligned}&\partial \Gamma _h \subset U_{\delta _0}(\partial \Gamma ) \end{aligned}$$

2.8 $$\begin{aligned}&\Vert \rho _\Gamma \Vert _{L^\infty ({\Gamma _h})} \lesssim h^{k+1} \end{aligned}$$

2.9 $$\begin{aligned}&\Vert n \circ p_\Gamma - n_h\Vert _{L^\infty ({\Gamma _h})} \lesssim h^k \end{aligned}$$

2.10 $$\begin{aligned}&\Vert \rho _{\partial \Gamma } \Vert _{L^\infty (\partial {\Gamma _h})} \lesssim h^{k+1} \end{aligned}$$

2.11 $$\begin{aligned}&\Vert \nu \circ p_{\partial \Gamma } - {\nu _{\Gamma _h}}\Vert _{L^\infty ({\Gamma _h})} \lesssim h^k \end{aligned}$$

Note that it follows that we also have the estimate

2.12 $$\begin{aligned} \Vert t_{\partial \Gamma }\circ p_{\partial \Gamma } - t_{\partial {\Gamma _h}}\Vert _{L^\infty (\partial {\Gamma _h})} \lesssim h^{k} \end{aligned}$$

for the unit tangent vectors $t_{\partial \Gamma }$ and $t_{\partial {\Gamma _h}}$ of $\partial \Gamma$ and $\partial {\Gamma _h}$.

**Finite element spaces** Let $V_h = V_h (\Gamma _h)$ be the space of parametric continuous piecewise polynomials of order *k* defined on $\mathcal {K}_h$, i.e.

2.13 $$\begin{aligned} V_h = \left\{ v \in C({\Gamma _h},\mathbb {R}){:}\; v|_K \in \widehat{V}_k \circ F_K^{-1}\right\} \end{aligned}$$

where $\widehat{V}_k = P_k(\widehat{K})$ is the space of polynomials of order less or equal to *k* defined on the reference triangle $\widehat{K}$ defined above. We study the approximation properties of $V_h$ in Sect. 3.4, where we define an interpolation operator and present associated interpolation error estimates.

### The finite element method

The finite element method for the boundary value problem (2.3)–(2.4) takes the form: find $u_h \in V_h$ such that

2.14 $$\begin{aligned} a_{{\Gamma _h}}(u_h, v) = l_{{\Gamma _h}}(v),\quad \forall v \in V_h \end{aligned}$$

where

2.15 $$\begin{aligned} a_{{\Gamma _h}}(v,w)&= (\nabla _{\Gamma _h}v,\nabla _{\Gamma _h}w)_{\Gamma _h} \nonumber \\&\quad - (\nu _{\partial {\Gamma _h}}\cdot \nabla _{\Gamma _h}v,w)_{\partial \Gamma _h} - (v, \nu _{\partial {\Gamma _h}}\cdot \nabla _{\Gamma _h}w)_{\partial \Gamma _h}\nonumber \\&\quad + \beta h^{-1}(v,w)_{\partial \Gamma _h} \end{aligned}$$

2.16 $$\begin{aligned} l_{{\Gamma _h}}(w)&= (f\circ p ,w)_{\Gamma _h} - (g \circ p_{\partial \Gamma } ,\nu _{\partial {\Gamma _h}}\cdot \nabla _{\Gamma _h}w)_{\partial \Gamma _h} + \beta h^{-1}(g\circ p_{\partial \Gamma }, w)_{\partial \Gamma _h} \end{aligned}$$

Here $\beta >0$ is a parameter, and *f* is extended from $\Gamma$ to $\Gamma \cup p(\Gamma _h) \subset \widetilde{\Gamma }$ in such a way that $f \in H^m(\Gamma \cup p({\Gamma _h}))$ and

2.17 $$\begin{aligned} \Vert f \Vert _{H^m(\Gamma \cup p({\Gamma _h}))} \lesssim \Vert f \Vert _{H^m(\Gamma )} \end{aligned}$$

where $m=0$ for $k=1$ and $m=1$ for $k\ge 2$.

#### Remark 2.2

Note that in order to prove optimal a priori error estimates for piecewise polynomials of order *k* we require $u\in H^{k+1}(\Gamma )$ and thus $f\in H^{k-1}(\Gamma )$. For $k=1$ we have $f\in L^2(\Gamma )$ and for $k\ge 2$ we require $f\in H^{k-1}(\Gamma )\subseteq H^1(\Gamma )$. Thus we conclude that (2.17) does not require any additional regularity compared to the standard situation. We will also see in Sect. 3.4 below that there indeed exists extensions of functions that preserve regularity.

## A priori error estimates

We derive a priori error estimates that take both the approximation of the geometry and the solution into account. The main new feature is that our analysis also takes the approximation of the boundary into account.

### Lifting and extension of functions

We collect some basic facts about lifting and extensions of functions, their derivatives, and related change of variable formulas, see for instance [5, 10, 11], for further details.

- For each function *v* defined on $\widetilde{\Gamma }$ we define the extension
  3.1 $$\begin{aligned} v^e = v \circ p \end{aligned}$$
  to $U_{\delta _{\widetilde{\Gamma }}}(\widetilde{\Gamma })$. For each function *v* defined on ${\Gamma _h}$ we define the lift to ${\Gamma _h^l}= p({\Gamma _h})\subset \widetilde{\Gamma }$ by
  3.2 $$\begin{aligned} v^l \circ p = v \end{aligned}$$
  Here and below we use the notation $\omega ^l = p(\omega )\subset \widetilde{\Gamma }$ for any subset $\omega \subset {\Gamma _h}$.
- The derivative $dp{:}\;T_x({\Gamma _h}) \rightarrow T_{p(x)}(\Gamma )$ of the closest point mapping $p{:}\;{\Gamma _h}\rightarrow \widetilde{\Gamma }$ is given by
  3.3 $$\begin{aligned} dp(x) = P_{\Gamma }(p(x)) P_{{\Gamma _h}}(x) + \rho (x) \mathcal {H}(x)P_{{\Gamma _h}}(x) \end{aligned}$$
  where $T_x(\Gamma )$ and $T_{p(x)}({\Gamma _h})$ are the tangent spaces to $\Gamma$ at $x \in \Gamma$ and to ${\Gamma _h}$ at $p(x)\in {\Gamma _h}$, respectively. Furthermore, $\mathcal {H}(x) = \nabla \otimes \nabla \rho (x)$ is the $\Gamma$ tangential curvature tensor which satisfies the estimate $\Vert \mathcal {H}\Vert _{L^\infty (U_{\delta }(\widetilde{\Gamma }))} \lesssim 1$, for some small enough $\delta >0$, see [14] for further details. We use *B* to denote a matrix representation of the operator *dp* with respect to an arbitrary choice of orthonormal bases in $T_x({\Gamma _h})$ and $T_{p(x)}(\Gamma )$. We also note that *B* is invertible.
- Gradients of extensions and lifts are given by
  3.4 $$\begin{aligned} \nabla _{\Gamma _h}v^e = B^T \nabla _\Gamma v, \quad \nabla _\Gamma v^l = B^{-T} \nabla _{\Gamma _h}v \end{aligned}$$
  where the gradients are represented as column vectors and the transpose $B^T{:}\;T_{p(x)}(\widetilde{\Gamma })\rightarrow T_x({\Gamma _h})$ is defined by $(B v,w)_{T_{p(x)}(\widetilde{\Gamma })} = (v, B^T w)_{T_x({\Gamma _h})}$, for all $v\in T_x({\Gamma _h})$ and $w\in T_{p(x)}(\widetilde{\Gamma })$.
- We have the following estimates
  3.5 $$\begin{aligned} \Vert B\Vert _{L^\infty (\Gamma _h)} \lesssim 1, \quad \Vert B^{-1}\Vert _{L^\infty (\Gamma )} \lesssim 1 \end{aligned}$$
- We have the change of variables formulas
  3.6 $$\begin{aligned} \int _{\omega ^l} g^l d\Gamma = \int _{\omega } g |B|d\Gamma _h \end{aligned}$$
  for a subset $\omega \subset {\Gamma _h}$, and
  3.7 $$\begin{aligned} \int _{\gamma ^l} g^l d\Gamma = \int _{\gamma } g |B_{\partial {\Gamma _h}}| d\Gamma _h \end{aligned}$$
  for a subset $\gamma \subset \partial \Gamma _h$. Here |*B*| denotes the absolute value of the determinant of *B* (recall that we are using orthonormal bases in the tangent spaces) and $|B_{\partial \Gamma _h}|$ denotes the norm of the restriction $B_{\partial {\Gamma _h}}{:}\; T_x(\partial {\Gamma _h}) \rightarrow T_{p(x)}(\partial {\Gamma _h^l})$ of *B* to the one dimensional tangent space of the boundary curve. We then have the estimates
  3.8 $$\begin{aligned} |\, |B|-1\,| \lesssim h^{k+1}, \quad | \, |B^{-1}| - 1 \,| \lesssim h^{k+1} \end{aligned}$$
  and
  3.9 $$\begin{aligned} |\, |B_{\partial \Gamma _h}| -1\,| \lesssim h^{k+1}, \quad | \, |B^{-1}_{\partial \Gamma _h}| - 1 \,| \lesssim h^{k+1} \end{aligned}$$
  Estimate (3.8) appear in several papers, see for instance [10]. Estimate (3.9) is less common but appears in papers on discontinuous Galerkin methods on surfaces, see [6, 9, 17]. For completeness we include a simple proof of (3.9). **Verification of** (3.9) Let $\gamma _{\Gamma _h}{:}\;[0,a) \rightarrow \partial {\Gamma _h}\subset \mathbb {R}^3$ be a parametrization of the curve $\partial {\Gamma _h}$ in $\mathbb {R}^3$, with *a* some positive real number. Then $p \circ \gamma _{\Gamma _h}(t)$, $t\in [0,a)$, is a parametrization of $\partial {\Gamma _h^l}$. We have
  3.10 $$\begin{aligned} |d_t \gamma _{\Gamma _h^l}|_{\mathbb {R}^3} = |d_t p \circ \gamma _{\Gamma _h}|_{\mathbb {R}^3} = |dp d_t \gamma _{\Gamma _h}|_{\mathbb {R}^3} = |B_{\partial {\Gamma _h}}||d_t \gamma _{\Gamma _h}|_{\mathbb {R}^3} \end{aligned}$$
  and since $d_t\gamma _{\Gamma _h} \in T_x(\Gamma _h)$ also
  3.11 $$\begin{aligned} |dp d_t \gamma _{\Gamma _h}|_{\mathbb {R}^3} - |d_t \gamma _{\Gamma _h}|_{\mathbb {R}^3}&=|(P_\Gamma + \rho \mathcal {H}) d_t \gamma _{\Gamma _h}|_{\mathbb {R}^3} - |d_t \gamma _{\Gamma _h}|_{\mathbb {R}^3} \end{aligned}$$
  3.12 $$\begin{aligned}&= \underbrace{|P_\Gamma d_t \gamma _{\Gamma _h}|_{\mathbb {R}^3} - |d_t \gamma _{\Gamma _h}|_{\mathbb {R}^3}}_{\bigstar = O(h^{2k})} + O(h^{k+1}) \end{aligned}$$
  Here we estimated $\bigstar$ by first using the identity
  3.13 $$\begin{aligned} | P_\Gamma d_t \gamma _{\Gamma _h}|^2&= | d_t \gamma _{\Gamma _h}-Q_\Gamma d_t \gamma _{\Gamma _h}|^2 \end{aligned}$$
  3.14 $$\begin{aligned}&= |d_t \gamma _{\Gamma _h}|^2 - 2 d_t \gamma _{\Gamma _h}\cdot Q_\Gamma d_t \gamma _{\Gamma _h}+ |Q_\Gamma d_t \gamma _{\Gamma _h}|^2 \end{aligned}$$
  3.15 $$\begin{aligned}&= |d_t \gamma _{\Gamma _h}|^2 - |Q_\Gamma d_t \gamma _{\Gamma _h}|^2 \end{aligned}$$
  3.16 $$\begin{aligned}&\ge ( 1- C h^{2k} ) | d_t \gamma _{\Gamma _h}|^2 \end{aligned}$$
  and then using the estimate $|(1 + \delta )^{1/2} - 1|\lesssim |\delta |$, for $-1 \le \delta$, to conclude that
  3.17 $$\begin{aligned} | | P_\Gamma d_t \gamma _{\Gamma _h}| - | d_t \gamma _{\Gamma _h}| | \lesssim h^{2k} |d_t\gamma _{\Gamma _h}| \end{aligned}$$
- The following equivalences of norms hold (uniformly in *h*)
  3.18 $$\begin{aligned}&\Vert v \Vert _{H^m\left( {\Gamma _h^l}\right) } \sim \Vert v^e \Vert _{H^m({\Gamma _h})},\quad m=0,1, \quad v \in H^m(\Gamma ) \end{aligned}$$
  3.19 $$\begin{aligned}&\Vert v^l \Vert _{H^m\left( {\Gamma _h^l}\right) } \sim \Vert v\Vert _{H^m({\Gamma _h})},\quad m=0,1, \quad v \in H^m({\Gamma _h}) \end{aligned}$$
  These estimates follow from the identities for the gradients (3.4), the uniform bounds (3.5) of *B*, and the bounds (3.8) for the determinant |*B*|.

### Norms

We define the norms

3.20 $$\begin{aligned} |||v |||^2_{{\Gamma _h}}= & {} \Vert \nabla _{\Gamma _h}v \Vert ^2_{{\Gamma _h}} + |||v |||^2_{\partial {\Gamma _h}}, \quad |||v |||^2_{\partial {\Gamma _h}} = h \Vert \nabla _{\Gamma _h}v \Vert ^2_{\partial {\Gamma _h}} + h^{-1} \Vert v \Vert ^2_{\partial {\Gamma _h}} \nonumber \\\end{aligned}$$

3.21 $$\begin{aligned} |||v |||^2_{{\Gamma _h^l}}= & {} \Vert \nabla _\Gamma v \Vert ^2_{{\Gamma _h^l}} + |||v |||^2_{\partial {\Gamma _h^l}}, \quad |||v |||^2_{\partial {\Gamma _h^l}} = h \Vert \nabla _\Gamma v \Vert ^2_{\partial {\Gamma _h^l}} + h^{-1} \Vert v \Vert ^2_{\partial {\Gamma _h^l}}\nonumber \\ \end{aligned}$$

Then the following equivalences hold

3.22 $$\begin{aligned} |||v^l |||_{\Gamma _h^l}\sim |||v |||_{\Gamma _h}, \quad |||v^l |||_{\partial {\Gamma _h^l}} \sim |||v |||_{\partial {\Gamma _h}}, \quad v \in H^1({\Gamma _h}) \end{aligned}$$

3.23 $$\begin{aligned} |||v |||_{\Gamma _h^l}\sim |||v^e |||_{\Gamma _h}, \quad |||v |||_{\partial {\Gamma _h^l}} \sim |||v^e |||_{\partial {\Gamma _h}}, \quad v \in H^1\left( {\Gamma _h^l}\right) \end{aligned}$$

#### Remark 3.1

We will see that it is convenient to have access to the norms $|||\cdot |||_{\partial {\Gamma _h}}$ and $|||\cdot |||_{\partial {\Gamma _h^l}}$, involving the boundary terms since that allows us to take advantage of stronger control of the solution to the dual problem in the vicinity of the boundary, which is used in the proof.

**Verification of** (3.22) In view of (3.19) it is enough to verify the equivalence $|||v^l |||_{\partial {\Gamma _h^l}} \sim |||v |||_{\partial {\Gamma _h}}$, between the boundary norms. First we have using a change of domain of integration from $\partial {\Gamma _h^l}$ to $\partial {\Gamma _h}$ and the bound (3.9),

3.24 $$\begin{aligned} h^{-1} \Vert v^l\Vert ^2_{\partial {\Gamma _h^l}} = h^{-1} ( v^l, v^l)_{\partial {\Gamma _h^l}} = h^{-1} ( v, v |B_{\partial {\Gamma _h}}|)_{\partial {\Gamma _h}} \sim h^{-1} \Vert v\Vert ^2_{\partial {\Gamma _h}} \end{aligned}$$

Next again changing domain of integration from $\partial {\Gamma _h^l}$ to $\partial {\Gamma _h}$, using the identity for the gradient (3.4), the uniform boundedness of $B^{-1}$, and (3.9) we obtain

3.25 $$\begin{aligned} h \left\| \nabla _\Gamma v^l \right\| ^2_{\partial {\Gamma _h^l}}&= h \left\| B^{-T}\nabla _{\Gamma _h}v \right\| ^2_{\partial {\Gamma _h^l}} = h\left( B^{-T}\nabla _{\Gamma _h}v, B^{-T}\nabla _{\Gamma _h}v \right) _{\partial {\Gamma _h^l}} \end{aligned}$$

3.26 $$\begin{aligned}&\quad = h\left( B^{-T}\nabla _{\Gamma _h}v, B^{-T}\nabla _{\Gamma _h}v |B_{\partial {\Gamma _h}}|\right) _{\partial {\Gamma _h}} \sim h\Vert \nabla _{\Gamma _h}v \Vert ^2_{\partial {\Gamma _h}} \end{aligned}$$

### Coercivity and continuity

Using standard techniques, see [19] or Chapter 14.2 in [16], we find that $a_{{\Gamma _h}}$ is coercive

3.27 $$\begin{aligned} |||v |||_{{\Gamma _h}}^2 \lesssim a_{{\Gamma _h}}(v,v) \quad \forall v \in V_h \end{aligned}$$

provided $\beta >0$ is large enough. Furthermore, it follows directly from the Cauchy–Schwarz inequality that $a_{\Gamma _h}$ is continuous

3.28 $$\begin{aligned} a_{\Gamma _h}(v,w) \lesssim |||v |||_{\Gamma _h}|||w |||_{\Gamma _h}\quad \forall v,w \in V_h + V^e({\Gamma _h}) \end{aligned}$$

where $V^e({\Gamma _h}) = \{w{:}\;{\Gamma _h}\rightarrow \mathbb {R}{:}\; w = v \circ p, v \in H^s(\Gamma ), s>3/2\}$. We also note that $l_{\Gamma _h}(v)\lesssim h^{-1/2} |||v |||_{\Gamma _h}$ for $v\in V_h$, and thus for fixed $h \in (0,h_0]$, existence and uniqueness of the solution $u_h\in V_h$ to the finite element problem (2.14) follows from the Lax–Milgram lemma.

### Extension and interpolation

**Extension** We note that there is an extension operator $E{:}\;H^s(\Gamma ) \rightarrow H^s(U_{\delta _0}(\Gamma )\cap \widetilde{\Gamma })$ such that

3.29 $$\begin{aligned} \Vert Ev \Vert _{H^s(U_{\delta _0}(\Gamma )\cap \widetilde{\Gamma })} \lesssim \Vert v \Vert _{H^s(\Gamma )}, \quad s\ge 0 \end{aligned}$$

This result follows by mapping to a reference neighborhood in $\mathbb {R}^2$ using a smooth local chart and then applying the extension theorem, see [13], and finally mapping back to the surface. For brevity we shall use the notation *v* for the extended function as well, i.e., $v=Ev$ on $U_{\delta _0}(\Gamma )\cap \widetilde{\Gamma }$. We can then extend *v* to $U_{\delta _0}(\Gamma )$ by using the closest point extension, we use the notation $v^e = (E v)^e$.

**Interpolation** We may now define the interpolation operator

3.30 $$\begin{aligned} \pi _h{:}\; L^2(\Gamma ) \ni v \mapsto \pi _{h,SZ} (E v)^e \in V_h \end{aligned}$$

where $\pi _{h,SZ}$ is a Scott–Zhang interpolation operator, see [22] and in particular the extension to triangulated surfaces in [8], without special treatment of the boundary condition. More precisely each node $x_i$ is associated with a triangle $S_i$ such that $x_i \in S_i$. Let $\{\varphi _{i,k}\}$ be the Lagrange basis on $S_i$ and let $\{\psi _{i,l} \}$ be the dual basis such that $(\varphi _{i,k},\psi _{j,l})_{S_i} = \delta _{i,j}$, and let $\psi _i$ be the dual basis function associated with node *i*. Then the nodal values are defined by

3.31 $$\begin{aligned} \pi _h v (x_i) = \left( (E v)^e, \psi _i\right) _{S_i} \end{aligned}$$

#### Remark 3.2

We need no particular adjustment of the interpolant at the boundary since we are using weak enforcement of the boundary conditions. In Remark 3.9 we consider strong boundary conditions and also use a Scott–Zhang interpolation operator which interpolates the boundary data at the boundary.

Then the following interpolation error estimate holds

3.32 $$\begin{aligned} \left\| v^e - \pi _h v^e \right\| _{H^m(K)} \lesssim h^{s-m}\Vert v \Vert _{H^s(\mathcal {N}^l_h(K))}, \quad 0\le m \le s \le k+1 \end{aligned}$$

where $\mathcal {N}^l_h(K)$ is the patch of elements which are node neighbors to *K* lifted onto $\Gamma _h^l \subset \widetilde{\Gamma }$. See Theorem 3.2 in [8] for a proof.

Using the trace inequality

3.33 $$\begin{aligned} \Vert w \Vert ^2_{\partial K} \lesssim h_K^{-1} \Vert w \Vert _K^2 + h_K \Vert \nabla _{\Gamma _h}w \Vert _K^2,\quad v \in H^1(K), K\in \mathcal {K}_h \end{aligned}$$

where $h_K \sim h$ is the diameter of element *K*, to estimate the boundary contribution in $|||\cdot |||_{{\Gamma _h}}$, followed by the interpolation estimate (3.32) and the stability of the extension operator (3.29), we conclude that

3.34 $$\begin{aligned} \left| \left| \left| v - \left( \pi _h v^e\right) ^l \right| \right| \right| _{\Gamma _h^l}\sim \left| \left| \left| v^e - \pi _h v^e \right| \right| \right| _{\Gamma _h}\lesssim h^k \Vert v \Vert _{H^{k+1}(\Gamma )} \end{aligned}$$

We will use the short hand notation $\pi _h^l v = (\pi _h v^e)^l$ for the lift of the interpolant. We refer to [10, 18] for further details on interpolation on triangulated surfaces.

### Strang Lemma

In order to formulate a Strang Lemma we first define auxiliary forms on ${\Gamma _h^l}$ corresponding to the discrete form on ${\Gamma _h}$ as follows

3.35 $$\begin{aligned} a_{{\Gamma _h^l}}\left( v,w\right)&= \left( \nabla _\Gamma v,\nabla _\Gamma w\right) _{\Gamma _h^l} \nonumber \\&\quad - \left( \nu _{\partial {\Gamma _h^l}}\cdot \nabla _\Gamma v,w\right) _{\partial \Gamma _h^l} - \left( v, \nu _{\partial {\Gamma _h^l}}\cdot \nabla _\Gamma w\right) _{\partial \Gamma _h^l} \nonumber \\&\quad + \beta h^{-1}\left( v,w\right) _{\partial \Gamma _h^l} \end{aligned}$$

3.36 $$\begin{aligned} l_{{\Gamma _h^l}}\left( w\right)&= \left( f,w\right) _{{\Gamma _h^l}} - \left( g \circ \widetilde{p}_{\partial \Gamma },\nu _{\partial {\Gamma _h^l}}\cdot \nabla _{\Gamma _h}w\right) _{\partial {\Gamma _h^l}} + \beta h^{-1}\left( g\circ \widetilde{p}_{\partial \Gamma }, w\right) _{\partial {\Gamma _h^l}} \end{aligned}$$

Here the mapping $\widetilde{p}_{\partial \Gamma }{:}\;\partial {\Gamma _h^l}\rightarrow \partial \Gamma$ is defined by the identity

3.37 $$\begin{aligned} \widetilde{p}_{\partial \Gamma }\circ p(x) = p_{\partial \Gamma }(x), \quad x \in \partial {\Gamma _h}\end{aligned}$$

Then we find that $\widetilde{p}_{\partial \Gamma }$ is a bijection since $p{:}\;\partial {\Gamma _h}\rightarrow \partial {\Gamma _h^l}$ and $p_{\partial \Gamma }{:}\; \partial {\Gamma _h}\rightarrow \partial \Gamma$ are bijections. Note that $a_{\Gamma _h^l}$, $l_{\Gamma _h^l}$, and $\widetilde{p}_{\partial \Gamma }$ are only used in the analysis and do not have to be implemented.

#### Lemma 3.1

With *u* the solution of (2.3–2.4) and $u_h$ the solution of (2.14) the following estimate holds

3.38 $$\begin{aligned} \left| \left| \left| u - u_h^l \right| \right| \right| _{\Gamma _h^l}&\lesssim \left| \left| \left| u - (\pi _h u)^l \right| \right| \right| _{\Gamma _h^l} \nonumber \\&\quad + \sup _{v \in V_h {\setminus } \{0\}} \frac{a_{{\Gamma _h}}( \pi _h u,v) - a_{{\Gamma _h^l}}\left( (\pi _h u)^l ,v^l\right) }{|||v |||_{{\Gamma _h}}} \nonumber \\&\quad + \sup _{v \in V_h {\setminus } \{0\}} \frac{l_{{\Gamma _h^l}}(v^l) - l_{{\Gamma _h}}(v)}{|||v |||_{{\Gamma _h}}} \nonumber \\&\quad + \sup _{v \in V_h {\setminus } \{0\}} \frac{a_{{\Gamma _h^l}}(u,v^l) - l_{{\Gamma _h^l}}(v^l)}{|||v |||_{{\Gamma _h}}} \end{aligned}$$

#### Remark 3.3

In (3.38) the first term on the right hand side is an interpolation error, the second and third terms account for the approximation of the surface $\Gamma$ by ${\Gamma _h}$ and can be considered as quadrature or geometric errors, finally the fourth term is a consistency error term which accounts for the approximation of the boundary of the surface.

#### Proof

We have

3.39 $$\begin{aligned} \left| \left| \left| u - u_h^l \right| \right| \right| _{{\Gamma _h^l}}&\lesssim \left| \left| \left| u - \left( \pi _h u^e\right) ^l \right| \right| \right| _{{\Gamma _h^l}} + \left| \left| \left| \left( \pi _h u^e\right) ^l- u_h^l \right| \right| \right| _{{\Gamma _h^l}} \end{aligned}$$

Using equivalence of norms (3.22) and coercivity of the bilinear form $a_h$ we have

3.40 $$\begin{aligned} \left| \left| \left| \left( \pi _h u^e\right) ^l- u_h^l \right| \right| \right| _{{\Gamma _h^l}} \sim \left| \left| \left| \pi _h u^e - u_h \right| \right| \right| _{\Gamma _h}&\lesssim \sup _{v \in V_h{\setminus }\{0\}} \frac{a_{\Gamma _h}\left( \pi _h u^e - u_h,v\right) }{\left| \left| \left| v \right| \right| \right| _{{\Gamma _h}}} \end{aligned}$$

Next we have the identity

3.41 $$\begin{aligned} a_{{\Gamma _h}}\left( \pi _h u^e - u_h,v\right)&= a_{{\Gamma _h}}\left( \pi _h u^e,v\right) - l_{\Gamma _h}\left( v\right) \end{aligned}$$

3.42 $$\begin{aligned}&= a_{{\Gamma _h}}\left( \pi _h u^e,v\right) - a_{{\Gamma _h^l}}\left( u,v^l\right) + l_{{\Gamma _h^l}}\left( v^l\right) - l_{\Gamma _h}\left( v\right) \nonumber \\&\quad + a_{{\Gamma _h^l}}\left( u,v^l\right) - l_{{\Gamma _h^l}}\left( v^l\right) \end{aligned}$$

3.43 $$\begin{aligned}&= \underbrace{a_{\Gamma _h}\left( \pi _h u^e,v\right) - a_{\Gamma _h^l}\left( \left( \pi _h u^e\right) ^l ,v^l\right) }_{I} + \underbrace{l_{{\Gamma _h^l}}\left( v^l\right) - l_{{\Gamma _h}}\left( v\right) }_{II} \nonumber \\&\quad + \underbrace{a_{\Gamma _h^l}\left( \left( \pi _h u^e\right) ^l - u,v^l\right) }_{III} + \underbrace{a_{{\Gamma _h^l}}\left( u,v^l\right) - l_{{\Gamma _h^l}}\left( v^l\right) }_{IV} \end{aligned}$$

where in (3.41) we used the equation (2.14) to eliminate $u_h$, in (3.42) we added and subtracted $a_{{\Gamma _h^l}}(u,v^l)$ and $l_{{\Gamma _h^l}}(v^l)$, in (3.43) we added and subtracted $a_{\Gamma _h^l}((\pi _h u^e)^l,v)$, and rearranged the terms. Combining (3.40) and (3.43) directly yields the Strang estimate (3.38). $\square$

### Estimate of the consistency error

In this section we derive an estimate for the consistency error, i.e., the fourth term on the right hand side in the Strang Lemma 3.1. First we derive an identity for the consistency error in Lemma 3.2 and then we prove two technical results in Lemma 3.3 and Lemma 3.4, and finally we give a bound of the consistency error in Lemma 3.5. In order to keep track of the error emanating from the boundary approximation we introduce the notation

3.44 $$\begin{aligned} \delta _h = \Vert \widetilde{\rho }_{\partial \Gamma } \Vert _{L^\infty (\partial {\Gamma _h^l})}\lesssim h^{k+1} \end{aligned}$$

where

3.45 $$\begin{aligned} \widetilde{\rho }_{\partial \Gamma }(x) = |\widetilde{p}_{\partial \Gamma }(x) - x|_{\mathbb {R}^3},\quad x \in {\Gamma _h^l}\end{aligned}$$

and we recall that $\widetilde{p}_{\partial \Gamma }$ is defined in (3.37). The estimate in (3.44) follows from the triangle inequality and the geometry approximation properties (2.8) and (2.10).

#### Lemma 3.2

Let *u* be the solution to (2.3–2.4), then the following identity holds

3.46 $$\begin{aligned} a_{\Gamma _h^l}\left( u,v^l\right) - l_{\Gamma _h^l}\left( v^l\right)&= -\left( f+\Delta _\Gamma u ,v^l\right) _{\Gamma _h^l {\setminus } \Gamma } \nonumber \\&\quad + \left( u\circ {\widetilde{p}_{\partial \Gamma }} - u, \nu _{\partial {\Gamma _h^l}}\cdot \nabla _\Gamma v^l\right) _{\partial {\Gamma _h^l}} - \beta h^{-1} \left( u\circ {\widetilde{p}_{\partial \Gamma }} - u, v^l\right) _{\partial {\Gamma _h^l}} \end{aligned}$$

for all $$v \in V_h$$.

#### Proof

For $v \in V_h$ we have using Green’s formula

3.47 $$\begin{aligned} \left( f,v^l\right) _{{\Gamma _h^l}}&= \left( f + \Delta _\Gamma u,v^l\right) _{{\Gamma _h^l}} - \left( \Delta _\Gamma u,v^l\right) _{{\Gamma _h^l}} \end{aligned}$$

3.48 $$\begin{aligned}&=\left( f + \Delta _\Gamma u ,v^l\right) _{\Gamma _h^l {\setminus } \Gamma } + \left( \nabla _\Gamma u,\nabla _\Gamma v^l\right) _{{\Gamma _h^l}} - \left( \nu _{\partial {\Gamma _h^l}}\cdot \nabla _\Gamma u,v^l\right) _{\partial {\Gamma _h^l}} \end{aligned}$$

3.49 $$\begin{aligned}&=\left( f+\Delta _\Gamma u ,v^l\right) _{\Gamma _h^l {\setminus } \Gamma } + a_{{\Gamma _h^l}}\left( u,v^l\right) + \left( u,\nu _{\partial {\Gamma _h^l}}\cdot \nabla _\Gamma v^l\right) _{\partial {\Gamma _h^l}} - \beta h^{-1} \left( u,v^l\right) _{\partial {\Gamma _h^l}} \end{aligned}$$

where we used the fact that $f+\Delta _\Gamma u = 0$ on $\Gamma$ and the definition (3.35) of $a_{\Gamma _h^l}$. Next using the boundary condition $u=g$ on $\partial \Gamma$ we conclude that

3.50 $$\begin{aligned} \left( f,v^l\right) _{{\Gamma _h^l}}&=\left( f+\Delta _\Gamma u ,v^l\right) _{\Gamma _h^l {\setminus } \Gamma } + a_{{\Gamma _h^l}}\left( u,v^l\right) + \left( u,\nu _{\partial {\Gamma _h^l}}\cdot \nabla _\Gamma v^l\right) _{\partial {\Gamma _h^l}} - \beta h^{-1} \left( u,v^l\right) _{\partial {\Gamma _h^l}} \nonumber \\&\quad - \left( u\circ \widetilde{p}_{\partial \Gamma }- g \circ \widetilde{p}_{\partial \Gamma }, \nu _{\partial {\Gamma _h^l}}\cdot \nabla _\Gamma v^l\right) _{\partial {\Gamma _h^l}} + \beta h^{-1} \left( u\circ \widetilde{p}_{\partial \Gamma }- g \circ \widetilde{p}_{\partial \Gamma }, v^l\right) _{\partial {\Gamma _h^l}} \end{aligned}$$

Rearranging the terms we obtain

3.51 $$\begin{aligned}&\left( f,v^l\right) _{\Gamma _h^l} - \left( g \circ \widetilde{p}_{\partial \Gamma }, \nu _{\partial {\Gamma _h^l}}\cdot \nabla _\Gamma v^l\right) _{\partial {\Gamma _h^l}} + \beta h^{-1} \left( g \circ \widetilde{p}_{\partial \Gamma },v^l\right) _{\partial {\Gamma _h^l}} \nonumber \\&\quad =\left( f+\Delta _\Gamma u ,v^l\right) _{\Gamma _h^l {\setminus } \Gamma } + a_{{\Gamma _h^l}}\left( u,v^l\right) \nonumber \\&\quad \quad - \left( u\circ \widetilde{p}_{\partial \Gamma }- u , \nu _{\partial {\Gamma _h^l}}\cdot \nabla _\Gamma v^l\right) _{\partial {\Gamma _h^l}} + \beta h^{-1} \left( u\circ \widetilde{p}_{\partial \Gamma }- u,v^l \right) _{\partial {\Gamma _h^l}} \end{aligned}$$

where the term on the left hand side is $l_{{\Gamma _h^l}}$ and the proof is complete. $\square$

#### Lemma 3.3

The following estimate holds

3.52 $$\begin{aligned} \Vert v \circ \widetilde{p}_{\partial \Gamma }- v \Vert _{\partial \Gamma _h^l} \lesssim \delta _h \Vert v \Vert _{H^2(\Gamma )},\quad v \in H^2(\Gamma ) \end{aligned}$$

where $$v|_{\partial {\Gamma _h^l}} = (E v)_{\partial {\Gamma _h^l}}$$.

#### Proof

For each $x \in {\Gamma _h^l}$ let $I_x$ be the line segment between *x* and $\widetilde{p}_{\partial \Gamma }(x)\in \partial \Gamma$, $t_x$ the unit tangent vector to $I_x$, and let $x(s) = (1 - s/{\rho _{\partial \Gamma }(x)})x + (s/\rho _{\partial \Gamma }(x)) \widetilde{p}_{\partial \Gamma }(x)$, $s\in [0,\rho _{\partial \Gamma }]$, be a parametrization of $I_x$. Then we have the following estimate

3.53 $$\begin{aligned} |v \circ \widetilde{p}_{\partial \Gamma }(x) - v(x)|&\lesssim \left| \int _0^{\rho _{\partial \Gamma }(x)} \nabla v^e(x(s))\cdot t_x ds\right| \end{aligned}$$

3.54 $$\begin{aligned}&\lesssim \Vert \nabla v^e \cdot t_x \Vert _{I_x} |\rho _{\partial \Gamma }(x)|^{1/2} \end{aligned}$$

3.55 $$\begin{aligned}&\lesssim \Vert (\nabla _\Gamma v)\circ p \Vert _{I_x} |\rho _{\partial \Gamma }(x)|^{1/2} \end{aligned}$$

3.56 $$\begin{aligned}&\lesssim \Vert \nabla _\Gamma v \Vert _{I_x^l} |\rho _{\partial \Gamma }(x)|^{1/2} \end{aligned}$$

where we used the following estimates: (3.54) the Cauchy–Schwarz inequality, (3.55) the chain rule to conclude that $\nabla v^e \cdot t_x = \nabla (v\circ p) \cdot t_x = ((\nabla _\Gamma v)\circ p) \cdot dp \cdot t_x$, and thus we have the estimate

3.57 $$\begin{aligned} \Vert \nabla v^e \cdot t_x \Vert _{I_x}\lesssim \Vert (\nabla _\Gamma v) \circ p \Vert _{I_x} \end{aligned}$$

since *dp* is uniformly bounded in $U_{\delta _0}(\widetilde{\Gamma })$, (3.56) changed the domain of integration from $I_x$ to $I_x^l = p(I_x) \subset \widetilde{\Gamma }$. Integrating over $\partial {\Gamma _h^l}$ gives

3.58 $$\begin{aligned} \Vert v \circ p_{\partial \Gamma }- v\Vert _{\partial {\Gamma _h^l}}^2&\lesssim \int _{\partial \Gamma _h^l} \Vert \nabla _\Gamma v \Vert ^2_{I_x^l} |\rho _{\partial \Gamma }(x)| dx \end{aligned}$$

3.59 $$\begin{aligned}&\lesssim \Vert \rho _{\partial \Gamma }\Vert _{L^\infty \left( {\Gamma _h^l}\right) } \int _{\partial \Gamma _h^l} \Vert \nabla _\Gamma v \Vert ^2_{I_x^l} dx \end{aligned}$$

3.60 $$\begin{aligned}&\lesssim \delta _h \int _{\partial \Gamma } \Vert \nabla _\Gamma v \Vert ^2_{I_{y}^l} d y \end{aligned}$$

3.61 $$\begin{aligned}&\lesssim \delta _h \Vert \nabla _\Gamma v \Vert ^2_{U_{\delta _h}(\partial \Gamma ) \cap \widetilde{\Gamma }} \end{aligned}$$

where we used the following estimates: (3.59) we used Hölder’s inequality, (3.60) we used the fact that $\Vert \rho _{\partial \Gamma }\Vert _{L^\infty ({\Gamma _h^l})} \lesssim \delta _h$ and changed domain of integration from $\partial {\Gamma _h^l}$ to $\partial \Gamma$, and (3.61) we integrated over a larger tubular neighborhood $U_{\delta _h}(\partial \Gamma )\cap \widetilde{\Gamma }=\{ x \in \widetilde{\Gamma }{:}\; |\rho _{\partial \Gamma }(x)|\lesssim \delta _h\}$ of $\partial \Gamma$ of thickness $2\delta _h$. We thus conclude that we have the estimate

3.62 $$\begin{aligned} \Vert v \circ p_{\partial \Gamma }- v\Vert _{\partial {\Gamma _h^l}}^2 \lesssim \delta _h \Vert \nabla _\Gamma v \Vert ^2_{U_{\delta _h}^l(\partial \Gamma ) \cap \widetilde{\Gamma }} \end{aligned}$$

In order to proceed with the estimates we introduce, for each $t\in [-\delta ,\delta ]$, with $\delta >0$ small enough, the surface

3.63 $$\begin{aligned} \Gamma _t = {\left\{ \begin{array}{ll} \Gamma \cup (U_t(\partial \Gamma )\cap \widetilde{\Gamma }) &{} t\ge 0 \\ \Gamma {\setminus } (U_{|t|}(\partial \Gamma )\cap \widetilde{\Gamma }) &{} t<0 \end{array}\right. } \end{aligned}$$

and its boundary $\partial \Gamma _t$. Starting from (3.62) and using Hölder’s inequality in the normal direction we obtain

3.64 $$\begin{aligned} \Vert v \circ p_{\partial \Gamma }- v \Vert _{\partial \Gamma _h^l}&\lesssim \delta _h \underbrace{\sup _{t\in [-\delta ,\delta ]} \Vert \nabla _\Gamma v \Vert _{{\partial \Gamma _t}}}_{\bigstar } \end{aligned}$$

3.65 $$\begin{aligned}&\lesssim \delta _h \Vert v \Vert _{H^2(\Gamma )} \end{aligned}$$

Here we estimated $\bigstar$ using a trace inequality

3.66 $$\begin{aligned} \sup _{t\in [-\delta ,\delta ]} C_t \Vert \nabla _\Gamma v \Vert _{{\partial \Gamma _t}}&\le \sup _{t\in [-\delta ,\delta ]} \Vert \nabla _\Gamma v \Vert _{H^1(\Gamma _t)} \end{aligned}$$

3.67 $$\begin{aligned}&\le \underbrace{\left( \sup _{t\in [-\delta ,\delta ]} C_t\right) }_{\lesssim 1} \Vert v \Vert _{H^2(\Gamma _{\delta })} \end{aligned}$$

3.68 $$\begin{aligned}&\lesssim \Vert v \Vert _{H^2(\Gamma )} \end{aligned}$$

where we used the stability (3.29) of the extension of *v* from $\Gamma _0 = \Gamma$ to $\Gamma _{\delta }$. To see that the constant $C_t$ is uniformly bounded for $t\in [-\delta ,\delta ]$, we may construct a diffeomorphism $F_t{:}\;\Gamma _0 \rightarrow \Gamma _t$ that also maps $\partial \Gamma _0$ onto $\Gamma _t$, which has uniformly bounded derivatives for $t\in [-\delta ,\delta ]$, see the construction in [7]. For $w \in H^1(\Gamma _t)$ we then have

3.69 $$\begin{aligned} \Vert w \Vert _{\partial \Gamma _t}&\lesssim \Vert w \circ F_t \Vert _{\partial \Gamma _0} \lesssim \Vert w \circ F_t \Vert _{H^1(\Gamma _0)} \lesssim \Vert w \Vert _{H^1(\Gamma _t)} \end{aligned}$$

where we used the uniform boundedness of first order derivatives of $F_t$ in the first and third inequality and applied a standard trace inequality on the fixed domain $\Gamma _0=\Gamma$ in the second inequality. $\square$

#### Lemma 3.4

The following estimates hold

3.70 $$\begin{aligned} \Vert v \Vert ^2_{{\Gamma _h^l}{\setminus } \Gamma }&\lesssim \delta _h \Vert v \Vert ^2_{\partial \Gamma } + \delta _h^2 \Vert \nabla _\Gamma v \Vert ^2_{{\Gamma _h^l}{\setminus } \Gamma } \end{aligned}$$

3.71 $$\begin{aligned} \Vert v \Vert ^2_{{\Gamma _h^l}{\setminus } \Gamma }&\lesssim \delta _h \Vert v \Vert ^2_{\partial {\Gamma _h^l}} + \delta _h^2 \Vert \nabla _\Gamma v \Vert ^2_{{\Gamma _h^l}{\setminus } \Gamma } \end{aligned}$$

for $$v \in H^1(U_{\delta _0}(\partial \Gamma ) \cap \widetilde{\Gamma })$$ and $$\delta _h \in (0,\delta _0]$$.

#### Proof

Using the same notation as in Lemma 3.3 and proceeding in the same way as in (3.53)–(3.56) we obtain, for each $y \in I_x$,

3.72 $$\begin{aligned} |v(y)|&\lesssim |v \circ \widetilde{p}_{\partial \Gamma }(x)| + \left| \int _0^{\rho _{\partial \Gamma }(y)} \nabla v^e(x(s))\cdot t_x ds\right| \end{aligned}$$

3.73 $$\begin{aligned}&\lesssim |v \circ \widetilde{p}_{\partial \Gamma }(x)| + \Vert \nabla _\Gamma v \Vert _{I_x^l} |\rho _{\partial \Gamma }(x)|^{1/2} \end{aligned}$$

3.74 $$\begin{aligned}&\lesssim |v \circ \widetilde{p}_{\partial \Gamma }(x)| + \delta _h^{1/2} \Vert \nabla _\Gamma v \Vert _{I_x^l} \end{aligned}$$

Integrating along $I_x$ we obtain

3.75 $$\begin{aligned} \int _{I_x} v^2(y) dy&\lesssim \int _{I_x} (|v \circ \widetilde{p}_{\partial \Gamma }(x)|^2 + \delta _h \Vert \nabla _\Gamma v \Vert ^2_{I_x^l}) dy \end{aligned}$$

3.76 $$\begin{aligned}&\lesssim \delta _h |v \circ \widetilde{p}_{\partial \Gamma }(x)|^2 + \delta _h^2 \Vert \nabla _\Gamma v \Vert ^2_{I_x^l} \end{aligned}$$

Finally, let $\partial \Gamma _{h,\text {out}}^l= \partial {\Gamma _h^l}{\setminus } \Gamma$, be the part of $\partial {\Gamma _h^l}$ that resides outside of $\Gamma$, then we have ${\Gamma _h^l}{\setminus } \Gamma = \cup _{x\in \partial \Gamma _{h,\text {out}}^l} I_x^l$, and using the estimate (3.76) together with suitable changes of variables of integration we obtain

3.77 $$\begin{aligned} \Vert v \Vert ^2_{{\Gamma _h^l}{\setminus } \Gamma }&\lesssim \int _{\partial \Gamma _{h,\text {out}}^l} \Big ( \delta _h |v \circ \widetilde{p}_{\partial \Gamma }(x)|^2 + \delta _h^2 \Vert \nabla _\Gamma v \Vert ^2_{I_x^l} \Big ) dx \end{aligned}$$

3.78 $$\begin{aligned}&\lesssim \delta _h \int _{\partial \Gamma _{h,\text {out}}^l} |v \circ \widetilde{p}_{\partial \Gamma }(x)|^2 dx + \delta _h^2 \int _{\partial \Gamma _{h,\text {out}}^l} \Vert \nabla _\Gamma v \Vert ^2_{I_x^l} dx \end{aligned}$$

3.79 $$\begin{aligned}&\lesssim \delta _h \Vert v \Vert _{\partial \Gamma }^2 + \delta _h^2 \Vert \nabla _\Gamma v \Vert ^2_{{\Gamma _h^l}{\setminus } \Gamma } \end{aligned}$$

Thus the first estimate follows. The second is proved using the same technique. $\square$

#### Lemma 3.5

Let *u* be the solution to (2.3–2.4), then the following estimates hold

3.80 $$\begin{aligned} \Big |a_{{\Gamma _h^l}}(u,v^l) - l_{{\Gamma _h^l}}(v^l)\Big |&\lesssim \delta _h \Vert u\Vert _{H^{k+1}(\Gamma )} \Big ( \Vert \nabla _\Gamma v^l \Vert _{\Gamma _h^l}+ h^{-1/2} |||v |||_{\partial {\Gamma _h^l}} \Big ) \end{aligned}$$

3.81 $$\begin{aligned}&\lesssim h^{-1/2} \delta _h \Vert u \Vert _{H^{k+1}(\Gamma )} |||v |||_{{\Gamma _h}} \quad \forall v \in V_h \end{aligned}$$

#### Remark 3.4

Here (3.80) will be used in the proof of the $L^2$ norm error estimate and (3.81) in the proof of the energy norm error estimate. As mentioned before we will use stronger control of the size of solution to the dual problem, which is used in the proof of the $L^2$ error estimate, close to the boundary to handle the additional factor of $h^{-1/2}$ multiplying $|||v |||_{\partial {\Gamma _h^l}}$.

#### Proof

Starting from the identity (3.46) and using the triangle and Cauchy–Schwarz inequalities we obtain

3.82 $$\begin{aligned} \Big |a_h^l(u,v^l) - l_h^l(v^l)\Big |&\lesssim \left\| f+\Delta _\Gamma u\right\| _{\Gamma _h^l{\setminus } \Gamma } \left\| v^l\right\| _{\Gamma _h^l {\setminus } \Gamma } \nonumber \\&\quad + \left\| u\circ \widetilde{p}_{\partial \Gamma }- u\right\| _{\partial \Gamma _h^l} \left\| \nu _{\partial {\Gamma _h^l}}\cdot \nabla _\Gamma v^l\right\| _{\partial \Gamma _h^l} \nonumber \\&\quad + h^{-1} \left\| u\circ \widetilde{p}_{\partial \Gamma }- u\right\| _{\partial {\Gamma _h^l}} \left\| v^l\right\| _{\partial \Gamma _h^l} \end{aligned}$$

3.83 $$\begin{aligned}&\lesssim \underbrace{\left\| f+\Delta _\Gamma u\right\| _{\Gamma _h^l{\setminus } \Gamma }}_{I} \underbrace{\left\| v^l\right\| _{\Gamma _h^l {\setminus } \Gamma }}_{II} \nonumber \\&\quad + \underbrace{\left\| u\circ \widetilde{p}_{\partial \Gamma }- u\right\| _{\partial \Gamma _h^l}}_{III} h^{-1/2} |||v^l |||_{\partial {\Gamma _h^l}} \end{aligned}$$

3.84 $$\begin{aligned}&\lesssim \underbrace{h \delta _h^{m+1/2}}_{IV\lesssim \delta _h} \left\| u\right\| _{H^{m+2}(\Gamma )} \Big ( \left\| \nabla _\Gamma v^l\right\| _{\Gamma _h^l}+ h^{-1/2}|||v^l |||_{\partial {\Gamma _h^l}}\Big ) \nonumber \\&\quad + \delta _h \left\| u \right\| _{H^2(\Gamma )} h^{-1/2} |||v^l|||_{\partial \Gamma _h^l} \end{aligned}$$

for all $v \in V_h$ and $m=0,1$. Here we used the following estimates.

**Term**$\varvec{I}$ For $m=0$ we have using the triangle inequality, followed by the stability (2.17) and (3.29) of the extensions of *f* and *u*,

3.85 $$\begin{aligned} \Vert f+\Delta _\Gamma u\Vert _{{\Gamma _h^l}{\setminus } \Gamma }&\lesssim \Vert f\Vert _{{\Gamma _h^l}{\setminus } \Gamma } + \Vert \Delta _\Gamma u\Vert _{{\Gamma _h^l}{\setminus } \Gamma } \lesssim \Vert f\Vert _{\Gamma } + \Vert u\Vert _{H^2(\Gamma )} \end{aligned}$$

3.86 $$\begin{aligned}&\quad \lesssim \Vert \Delta _\Gamma u\Vert _{\Gamma } + \Vert u\Vert _{H^2(\Gamma )} \lesssim \Vert u\Vert _{H^2(\Gamma )} \end{aligned}$$

where we finally replaced *f* by $-\Delta _\Gamma u$ on $\Gamma$.

For $m=1$ we note that it follows from assumption (2.17) that $f + \Delta _\Gamma u \in H^1({\Gamma _h^l}\cup \Gamma )$ and $f+\Delta _\Gamma u = 0$ on $\Gamma$, which implies $f+\Delta _\Gamma u = 0$ on $\partial \Gamma$ since the trace is well defined. We may therefore apply the Poincaré estimate (3.70) to extract a power of $\delta _h$, as follows

3.87 $$\begin{aligned}&\Vert f+\Delta _\Gamma u\Vert _{\Gamma _h^l{\setminus } \Gamma } \lesssim \delta _h \Vert f+\Delta _\Gamma u\Vert _{H^1({\Gamma _h^l}{\setminus } \Gamma )} \lesssim \delta _h ( \Vert f \Vert _{H^1(\Gamma \cup {\Gamma _h^l})} + \Vert \Delta _\Gamma u \Vert _{H^1(\Gamma \cup {\Gamma _h^l})} ) \end{aligned}$$

3.88 $$\begin{aligned}&\quad \lesssim \delta _h ( \Vert f \Vert _{H^1(\Gamma )} + \Vert u \Vert _{H^3(\Gamma )} ) \lesssim \delta _h ( \Vert \Delta _\Gamma u \Vert _{H^1(\Gamma )} + \Vert u \Vert _{H^3(\Gamma )} ) \lesssim \delta _h \Vert u \Vert _{H^{3}(\Gamma )} \end{aligned}$$

where again we used the triangle inequality, the stability (2.17) and (3.29), and finally replaced *f* by $-\Delta _\Gamma u$ on $\Gamma$.

**Term**$\varvec{I}\varvec{I}$ We used the Poincaré estimate (3.71) as follows

3.89 $$\begin{aligned} \left\| v^l \right\| ^2_{\Gamma _h^l {\setminus } \Gamma }&\lesssim \delta _h^2 \left\| \nabla _\Gamma v^l \right\| ^2_{\Gamma _h^l {\setminus } \Gamma } + \delta _h \left\| v^l \right\| ^2_{\partial {\Gamma _h^l}} \end{aligned}$$

3.90 $$\begin{aligned}&\lesssim \delta _h^2 \left\| \nabla _\Gamma v^l \right\| ^2_{\Gamma _h^l {\setminus } \Gamma } + h^2 \delta _h h^{-2}\left\| v^l \right\| ^2_{\partial {\Gamma _h^l}} \end{aligned}$$

3.91 $$\begin{aligned}&\lesssim \underbrace{(\delta _h^2 + h^2 \delta _h )}_{\lesssim h^2 \delta _h} \Big (\left\| \nabla _\Gamma v^l \right\| ^2_{\Gamma _h^l {\setminus } \Gamma } +h^{-2}\left\| v^l \right\| ^2_{\partial {\Gamma _h^l}}\Big ) \end{aligned}$$

3.92 $$\begin{aligned}&\lesssim h^2 \delta _h \Big ( \left\| \nabla _\Gamma v^l\right\| ^2_{\Gamma _h^l}+ h^{-1}|||v^l |||^2_{\partial {\Gamma _h^l}}\Big ) \end{aligned}$$

**Term**$\varvec{I}\varvec{I}\varvec{I}$ We used the bound (3.52) to estimate $\Vert u\circ \widetilde{p}_{\partial \Gamma }- u\Vert _{\partial {\Gamma _h^l}}$.

**Factor**$\varvec{I}\varvec{V}$ We note that since $\delta _h \lesssim h^2$ and $h \in (0,h_0]$ we have $h\delta _h^{m+1/2} \lesssim \delta _h$ for $m=0$ and $m=1$.

This concludes the proof of estimate (3.80). Estimate (3.81) follows by a direct estimate of the right hand side of (3.80). $\square$

### Estimates of the quadrature errors

#### Lemma 3.6

The following estimates hold

3.93 $$\begin{aligned} \left\| |B|B^{-1} B^{-T} - P_{{\Gamma _h}} \right\| _{L^\infty ({\Gamma _h})} \lesssim h^{k+1} \end{aligned}$$

and

3.94 $$\begin{aligned} \left\| |B_{\partial {\Gamma _h}}|B^{-1} \nu _{\partial {\Gamma _h^l}}- \nu _{\partial {\Gamma _h}}\right\| _{L^\infty (\partial {\Gamma _h})} \lesssim h^{k+1} \end{aligned}$$

#### Remark 3.5

Recall that $B(x){:}\;T_x({\Gamma _h}) \rightarrow T_{p(x)}(\Gamma )$ and $B^T(x){:}\;T_{p(x)}(\Gamma )\rightarrow T_x({\Gamma _h})$ and therefore $B^{-1} B^{-T}{:}\;T_x({\Gamma _h}) \rightarrow T_x({\Gamma _h})$. In (3.93) we thus estimate the deviation of $|B|B^{-1} B^{-T}$ from the identity $P_{\Gamma _h}$ operator on $T_x({\Gamma _h})$.

#### Proof

(3.93): We have the estimate

3.95 $$\begin{aligned} \left\| |B|B^{-1} B^{-T} - P_{{\Gamma _h}} \right\| _{L^\infty ({\Gamma _h})}&\lesssim \left\| |B|P_\Gamma - B P_{{\Gamma _h}} B^T\right\| _{L^\infty (\Gamma )} \end{aligned}$$

3.96 $$\begin{aligned}&\lesssim \left\| P_\Gamma - P_\Gamma P_{{\Gamma _h}}P_\Gamma \right\| _{L^\infty (\Gamma )} + h^{k+1} \end{aligned}$$

where we used the uniform boundedness of $B^{-1}$, the identity $|B|= 1 + O(h^{k+1})$, see (3.8), and, the identity $B = P_\Gamma + O(h^{k+1})$, see (3.3). Next we have the identity

3.97 $$\begin{aligned} P_\Gamma - P_\Gamma P_{{\Gamma _h}}P_\Gamma&= P_\Gamma (I-P_{{\Gamma _h}}) P_\Gamma = P_\Gamma Q_{{\Gamma _h}}P_\Gamma = (P_\Gamma n_h)\otimes (P_\Gamma n_h) \end{aligned}$$

and thus

3.98 $$\begin{aligned} \Vert P_\Gamma - P_\Gamma P_{{\Gamma _h}}P_\Gamma \Vert _{L^\infty (\Gamma )}&\lesssim \Vert P_\Gamma n_h\Vert ^2_{L^\infty (\Gamma )} \lesssim \Vert n_h - n\Vert ^2_{L^\infty (\Gamma )} \lesssim h^{2k} \end{aligned}$$

which together with (3.96) concludes the proof.

(3.94): Using the uniform boundedness of $B^{-1}$ we obtain

3.99 $$\begin{aligned} \Vert |B_{\partial {\Gamma _h}}|B^{-1} \nu _{\partial {\Gamma _h^l}}- \nu _{\partial {\Gamma _h}}\Vert _{L^\infty ({\Gamma _h})} \lesssim \left\| |B_{\partial {\Gamma _h}}|\nu _{\partial {\Gamma _h^l}}- B \nu _{\partial {\Gamma _h}}\right\| _{L^\infty \left( {\Gamma _h^l}\right) } \end{aligned}$$

Next let $t_{\partial {\Gamma _h}}$ be the unit tangent vector to $\partial {\Gamma _h}$ and $t_{\partial {\Gamma _h^l}}$ the unit tangent vector to $\partial {\Gamma _h^l}$, oriented in such a way that $\nu _{\partial {\Gamma _h}}= t_{\partial {\Gamma _h}}\times n_h$ and $\nu _{\partial {\Gamma _h^l}}= t_{\partial {\Gamma _h^l}}\times n$. We then have

3.100 $$\begin{aligned} B\nu _{\partial {\Gamma _h}}&= (P_\Gamma P_{{\Gamma _h}}+ \rho \mathcal {H})\nu _{\partial {\Gamma _h}}\end{aligned}$$

3.101 $$\begin{aligned}&=P_\Gamma ( t_{\partial {\Gamma _h}}\times n_h ) + O(h^{k+1}) \end{aligned}$$

3.102 $$\begin{aligned}&= P_\Gamma ((P_\Gamma +Q_\Gamma )t_{\partial {\Gamma _h}}\times (P_\Gamma +Q_\Gamma )n_h) + O(h^{k+1}) \end{aligned}$$

3.103 $$\begin{aligned}&= P_\Gamma (P_\Gamma t_{\partial {\Gamma _h}}\times Q_\Gamma n_h + \underbrace{Q_\Gamma t_{\partial {\Gamma _h}}\times P_\Gamma n_h}_{O(h^{2k})}) + O(h^{k+1}) \end{aligned}$$

3.104 $$\begin{aligned}&= P_\Gamma t_{\partial {\Gamma _h}}\times Q_\Gamma n_h + O(h^{k+1}) \end{aligned}$$

where we used the fact that $P_\Gamma t_{\partial {\Gamma _h}}\times P_\Gamma n_h$ is normal to $\widetilde{\Gamma }$ and $Q_\Gamma t_{\partial {\Gamma _h}}\times Q_\Gamma n_h = 0$ since the vectors are parallel. Using (3.104) and adding and subtracting a suitable term we obtain

3.105 $$\begin{aligned} |B_{\partial {\Gamma _h}}|\nu _{\partial {\Gamma _h^l}}- B \nu _{\partial {\Gamma _h}}&= |B_{\partial {\Gamma _h}}|t_{\partial {\Gamma _h^l}}\times n - P_\Gamma t_{\partial {\Gamma _h}}\times Q_\Gamma n_h + O(h^{k+1}) \end{aligned}$$

3.106 $$\begin{aligned}&=\underbrace{(|B_{\partial {\Gamma _h}}|t_{\partial {\Gamma _h^l}}- P_\Gamma t_{\partial {\Gamma _h}})}_{I=O(h^{k+1})}\times n + P_\Gamma t_{\partial {\Gamma _h^l}}\times \underbrace{(n - Q_\Gamma n_h)}_{II=O(h^{2k})} + O(h^{k+1}) \end{aligned}$$

3.107 $$\begin{aligned}&=O(h^{k+1}) \end{aligned}$$

Here we used the estimates: (*I*) We have $|B_{\partial {\Gamma _h}}|t_{\partial {\Gamma _h^l}}= B t_{\partial {\Gamma _h}}$ and thus

3.108 $$\begin{aligned} |B_{\partial {\Gamma _h}}|t_{\partial {\Gamma _h^l}}- P_\Gamma t_{\partial {\Gamma _h}}= (B - P_\Gamma ) t_{\partial {\Gamma _h}}= \rho \mathcal {H} t_{\partial {\Gamma _h}}= O(h^{k+1}) \end{aligned}$$

(*II*) $$n - Q_\Gamma n_h = (1 - n\cdot n_h) {n} = 2^{-1} |n - n_h|^2 {n} = O(h^{2k})$$. $$\square $$

#### Lemma 3.7

The following estimates hold

3.109 $$\begin{aligned}&\Big |a_{{\Gamma _h^l}}(v^l,w^l) - a_{{\Gamma _h}}(v,w) \Big | \nonumber \\&\quad \lesssim h^{k+1} \Big (\Vert \nabla _{\Gamma _h}v \Vert _{{\Gamma _h}} + h^{1/2} |||v |||_{\partial {\Gamma _h}} \Big )\Big ( \Vert \nabla _{\Gamma _h}w \Vert _{{\Gamma _h}} + h^{-1/2} |||w |||_{\partial {\Gamma _h}} \Big ) \end{aligned}$$

3.110 $$\begin{aligned}&\quad \lesssim h^{k+1/2} |||v |||_{{\Gamma _h}} |||w |||_{{\Gamma _h}} \quad \forall v, w \in V_h \end{aligned}$$

and

3.111 $$\begin{aligned} \Big | l_{{\Gamma _h^l}}(v^l) - l_{{\Gamma _h}}(v)\Big |&\lesssim h^{k+1} \Big ( \Vert f\Vert _\Gamma + \Vert g \Vert _{\partial \Gamma } \Big ) \Big (\Vert \nabla _{\Gamma _h}v \Vert _{\Gamma _h}+ h^{-1/2}|||v |||_{\partial {{\Gamma _h}}} \Big ) \end{aligned}$$

3.112 $$\begin{aligned}&\lesssim h^{k+1/2} \Big ( \Vert f\Vert _\Gamma + \Vert g\Vert _{\partial \Gamma } \Big ) |||v |||_{\Gamma _h}\quad \forall v\in V_h \end{aligned}$$

#### Remark 3.6

In fact the estimate (3.110) holds also with the factor $h^{k+1}$, which is easily seen in the proof below. However, (3.110) is only used in the proof of the energy norm error estimate which is of order $h^k$ so there is no loss of order. We have chosen this form since it is analogous with the estimates of the right hand side (3.111)–(3.112).

#### Remark 3.7

We note that the estimates in Lemma 3.7 have similar form as the estimates in Lemma 3.5, which are adjusted to fit the $L^2$ and energy norm estimates.

#### Proof

(3.109)–(3.110): Starting from the definitions of the forms (2.15) and (3.35) we obtain

3.113 $$\begin{aligned} a_{{\Gamma _h^l}}\left( v^l,w^l\right) - a_{{\Gamma _h}}\left( v,w\right)&= \left( \nabla _\Gamma v^l,\nabla _\Gamma w^l\right) _{\Gamma _h^l} - \left( \nabla _{\Gamma _h}v,\nabla _{\Gamma _h}w\right) _{\Gamma _h} \nonumber \\&\quad - \left( \nu _{\partial {\Gamma _h^l}}\cdot \nabla _\Gamma v^l,w^l\right) _{\partial \Gamma _h^l} +\left( \nu _{\partial {\Gamma _h}}\cdot {\nabla _{\Gamma _h}} v,w\right) _{\partial \Gamma _h} \nonumber \\&\quad - \left( v^l, \nu _{\partial {\Gamma _h^l}}\cdot \nabla _\Gamma w^l\right) _{\partial \Gamma _h^l} + \left( v, \nu _{\partial {\Gamma _h}}\cdot {\nabla _{\Gamma _h}} w\right) _{\partial \Gamma _h} \nonumber \\&\quad + \beta h^{-1}\Big ( (v^l,w^l)_{\partial {\Gamma _h^l}} - (v,w)_{\partial \Gamma _h} \Big ) \end{aligned}$$

3.114 $$\begin{aligned}&= I + II + III + III \end{aligned}$$

**Term**$\varvec{I}$ We have the estimates

3.115 $$\begin{aligned} |I|&= \Big |\left( B^{-T} \nabla _{\Gamma _h}v, B^{-T} \nabla _{\Gamma _h}w {|B|} \right) _{{\Gamma _h}} - \left( \nabla _{\Gamma _h}v, \nabla _{\Gamma _h}w\right) _{{\Gamma _h}}\Big | \end{aligned}$$

3.116 $$\begin{aligned}&=\Big |\left( \left( |B|B^{-1} B^{-T} - P_{{\Gamma _h}}\right) \nabla _{\Gamma _h}v, \nabla _{\Gamma _h}w \right) _{{\Gamma _h}}\Big | \end{aligned}$$

3.117 $$\begin{aligned}&\lesssim h^{k+1} \Vert \nabla _{\Gamma _h}v \Vert _{{\Gamma _h}} \Vert \nabla _{\Gamma _h}w \Vert _{{\Gamma _h}} \end{aligned}$$

where we used the estimate (3.93).

**Terms**$\varvec{I}\varvec{I}$ and $\varvec{I}\varvec{I}\varvec{I}$ Terms *II* and *III* have the same form and may be estimated as follows

3.118 $$\begin{aligned} |II|&= \Big |\left( \nu _{\partial {\Gamma _h^l}}\cdot \nabla _\Gamma v^l,w^l\right) _{\partial \Gamma _h^l} {-}\left( \nu _{\partial {\Gamma _h}}\cdot {\nabla _{\Gamma _h}} v,w\right) _{\partial \Gamma _h}\Big | \end{aligned}$$

3.119 $$\begin{aligned}&=\Big |\left( \nu _{\partial {\Gamma _h^l}}\cdot B^{-T} \nabla _{\Gamma _h}v,w |B_{\partial {\Gamma _h}}|\right) _{{\partial \Gamma _h}} {-} \left( \nu _{\partial {\Gamma _h}}\cdot {\nabla _{\Gamma _h}},w\right) _{\partial \Gamma _h}\Big | \end{aligned}$$

3.120 $$\begin{aligned}&=\Big |\left( \left( |B_{\partial {\Gamma _h}}|B^{-1} \nu _{\partial {\Gamma _h^l}}- \nu _{\partial {\Gamma _h}}\right) {\cdot } \nabla _{\Gamma _h}v,w \right) _{{\partial \Gamma _h}} \Big | \end{aligned}$$

3.121 $$\begin{aligned}&\le \left\| |B_{\partial {\Gamma _h}}|B^{-1} \nu _{\partial {\Gamma _h^l}}- \nu _{\partial {\Gamma _h}}\right\| _{L^\infty \left( \partial {\Gamma _h}\right) } \Vert \nabla _{\Gamma _h}v \Vert _{\partial {\Gamma _h}} \Vert w \Vert _{\partial {\Gamma _h}} \end{aligned}$$

3.122 $$\begin{aligned}&\lesssim h^{k+1} h^{1/2}|||v |||_{{{\Gamma _h}}} h^{-1/2}|||w |||_{\partial {\Gamma _h}} \end{aligned}$$

where we used (3.94) and the inverse estimate

3.123 $$\begin{aligned} h \Vert \nabla _{\Gamma _h}v \Vert ^2_{{\partial }{\Gamma _h}} \lesssim \Vert \nabla _{\Gamma _h}v \Vert ^2_{\mathcal {K}_h({\Gamma _h})} \lesssim \Vert \nabla _{\Gamma _h}v \Vert ^2_{{\Gamma _h}} \end{aligned}$$

for all $v \in V_h$. Thus we conclude that

3.124 $$\begin{aligned} |II| + |III| \lesssim h^{k+1} h^{1/2}|||v |||_{{{\Gamma _h}}} h^{-1/2}|||w |||_{\partial {\Gamma _h}} \end{aligned}$$

**Term **$\varvec{I}\varvec{V}$ We have

3.125 $$\begin{aligned} |IV|&= \beta h^{-1}\Big | (v^l,w^l)_{\partial {\Gamma _h^l}} - (v,w)_{\partial {\Gamma _h}}\Big | \end{aligned}$$

3.126 $$\begin{aligned}&= \beta h^{-1}\Big |((|B_{\partial {\Gamma _h}}|- 1) v,w)_{\partial {\Gamma _h}}\Big | \end{aligned}$$

3.127 $$\begin{aligned}&\lesssim h^{-1}\Vert |B_{\partial {\Gamma _h}}|- 1\Vert _{L^\infty (\partial {\Gamma _h}{)}} \Vert v\Vert _{\partial {\Gamma _h}} \Vert w\Vert _{\partial {\Gamma _h}} \end{aligned}$$

3.128 $$\begin{aligned}&\lesssim h^{k+1} h^{1/2}|||v |||_{\partial {\Gamma _h}} h^{-1/2}|||w |||_{\partial {\Gamma _h}} \end{aligned}$$

Estimate (3.110) follows by a direct estimate of the right hand side of (3.109).

(3.111) and (3.112): We have

3.129 $$\begin{aligned} \Big |l_{\Gamma _h^l}\big (w^l\big ) - l_{\Gamma _h}\big (w\big )\Big |&= \Big |\big (f,w^l\big )_{{\Gamma _h^l}} - \big (f\circ p_\Gamma ,w\big )_{\Gamma _h}\nonumber \\&\quad - \big (g \circ \widetilde{p}_{\partial \Gamma },\nu _{\partial {\Gamma _h^l}}\cdot \nabla _\Gamma w^l\big )_{\partial {\Gamma _h^l}} + \big (g \circ p_{\partial \Gamma } ,\nu _{\partial {\Gamma _h}}\cdot \nabla _{\Gamma _h}w\big )_{\partial {\Gamma _h}} \nonumber \\&\quad + \beta h^{-1}\big (g\circ \widetilde{p}_{\partial \Gamma }, w^l\big )_{\partial {\Gamma _h^l}} - \beta h^{-1}\big (g\circ p_{\partial \Gamma }, w\big )_{\partial {\Gamma _h}} \Big | \end{aligned}$$

3.130 $$\begin{aligned}&\le \Big |\big ({|B|} -1\big ) f\circ p_\Gamma ,w\big )_{\Gamma _h}\Big | \nonumber \\&\quad + \Big | \big (g \circ p_{\partial \Gamma } , \big ({|B_{\partial {\Gamma _h}}|B^{-1}} \nu _{\partial {\Gamma _h^l}}- \nu _{\partial {\Gamma _h}}\big ) \cdot \nabla _{\Gamma _h}w\big )_{\partial {\Gamma _h}}\Big | \nonumber \\&\quad + \beta h^{-1}\Big |\big (\big ({|B_{\partial {\Gamma _h}}|}-1\big )g\circ p_{\partial \Gamma }, w\big )_{\partial {\Gamma _h}} \Big | \end{aligned}$$

3.131 $$\begin{aligned}&\lesssim h^{k+1} \Vert f \Vert _\Gamma \Vert w\Vert _{\Gamma _h}+ h^{k+1} \Vert g \Vert _{\partial \Gamma } \Vert \nabla _{\Gamma _h}w\Vert _{{\partial }{\Gamma _h}} + h^{k}\Vert g \Vert _{\partial \Gamma } \Vert w\Vert _{\partial {\Gamma _h}} \end{aligned}$$

where we used (3.8), (3.94) and (3.9). Next using the Poincaré estimate

3.132 $$\begin{aligned} \Vert w \Vert _{\Gamma _h}\lesssim \Vert \nabla _{\Gamma _h}w \Vert _{\Gamma _h}+ \Vert w \Vert _{\partial {\Gamma _h}} \lesssim \Vert \nabla _{\Gamma _h}w \Vert _{\Gamma _h}+ h^{1/2}|||w |||_{\partial {\Gamma _h}} \end{aligned}$$

we obtain

3.133 $$\begin{aligned} \Big |l_{\Gamma _h^l}(w^l) - l_{\Gamma _h}(w)\Big |&\lesssim h^{k+1} \Vert f \Vert _\Gamma \Vert w\Vert _{\Gamma _h}\nonumber \\&\quad + h^{k+1} \Vert g \Vert _{\partial \Gamma } h^{-1/2}|||w |||_{{ \partial {\Gamma _h}}} + {h^{k} \Vert g \Vert _{\partial \Gamma } h^{1/2}|||w |||_{\partial {\Gamma _h}}} \end{aligned}$$

3.134 $$\begin{aligned}&\lesssim h^{k+1} \Vert f \Vert _\Gamma \Big ( \Vert \nabla _{\Gamma _h}w \Vert _{\Gamma _h}+ h^{1/2}|||w |||_{\partial {\Gamma _h}} \Big ) \nonumber \\&\quad + h^{k+1} \Vert g \Vert _{\partial \Gamma } h^{-1/2}|||w |||_{{ \partial {\Gamma _h}}} \end{aligned}$$

3.135 $$\begin{aligned}&\lesssim h^{k+1}\Big ( \Vert f \Vert _\Gamma + \Vert g \Vert _{\partial \Gamma }\Big ) \Big ( \Vert \nabla _{\Gamma _h}w \Vert _{\Gamma _h}+ h^{-1/2}|||w |||_{\partial {\Gamma _h}} \Big ) \end{aligned}$$

3.136 $$\begin{aligned}&\lesssim h^{k+1/2}\Big ( \Vert f \Vert _\Gamma + \Vert g \Vert _{\partial \Gamma }\Big ) |||w |||_{\Gamma _h}\end{aligned}$$

which are the desired estimates. $\square$

### Error estimates

With the Strang Lemma 3.1 and the estimates for the interpolation, quadrature, and consistency errors at hand, we are now prepared to prove the main a priori error estimates.

#### Theorem 3.1

With *u* the solution of (2.3)–(2.4) and $u_h$ the solution of (2.14) the following estimate holds

3.137 $$\begin{aligned} |||u - u_h^l |||_{{\Gamma _h^l}} \lesssim h^k \Big (\Vert u \Vert _{H^{k+1}(\Gamma )} + \Vert f\Vert _{\Gamma } + \Vert g\Vert _{\partial \Gamma } \Big ) \end{aligned}$$

#### Proof

Starting from the Strang Lemma and using the interpolation estimate (3.34), the quadrature error estimates (3.110) and (3.112), and the consistency error estimate (3.81), we obtain

3.138 $$\begin{aligned} |||u - u_h^l |||_{{\Gamma _h^l}}&\lesssim h^k \Vert u \Vert _{H^{k+1}(\Gamma )} + h^{k+1/2} |||\pi _h u^e |||_{{\Gamma _h}} + h^{k+1/2} \Big ( \Vert f\Vert _{\Gamma } + \Vert g \Vert _{\partial \Gamma } \Big ) \nonumber \\&\quad + h^{-1/2}\delta _h \Vert u \Vert _{H^2(\Gamma )} \end{aligned}$$

3.139 $$\begin{aligned}&\lesssim h^k \Vert u \Vert _{H^{k+1}(\Gamma )} + h^{k+1/2} \Big ( \Vert f\Vert _{\Gamma } + \Vert g \Vert _{\partial \Gamma } \Big ) \nonumber \\&\quad + h^{k+1/2} \Vert u \Vert _{H^2(\Gamma )} \end{aligned}$$

Here, in (3.139), we used the estimate

3.140 $$\begin{aligned} |||\pi _h u^e |||_{{\Gamma _h}}&\lesssim |||\pi _h u^e - u^e |||_{{\Gamma _h}} + |||u^e |||_{{\Gamma _h}} \end{aligned}$$

3.141 $$\begin{aligned}&\lesssim h^k\Vert u\Vert _{H^{k+1}(\Gamma )} + h^{-1/2} \Vert u \Vert _{H^2(\Gamma )} \end{aligned}$$

where, in (3.141), we used the interpolation estimate (3.34) to estimate the first term and a trace inequality to estimate the second term, and finally the inequality $h^{-1/2} \delta _h \lesssim h^{k+1/2}$. Thus the proof is complete since $k\ge 1$ and $h\in (0,h_0]$. $\square$

#### Theorem 3.2

With *u* the solution of (2.3–2.4) and $u_h$ the solution of (2.14) the following estimate holds

3.142 $$\begin{aligned} \Vert u - u_h^l \Vert _{{\Gamma _h^l}} \lesssim h^{k+1} \Big (\Vert u \Vert _{H^{k+1}(\Gamma )} + \Vert f\Vert _{\Gamma } + \Vert g\Vert _{\partial \Gamma }\Big ) \end{aligned}$$

#### Proof

Let $\phi \in H^1_0(\Gamma )$ be the solution to the dual problem

3.143 $$\begin{aligned} a(v,\phi ) = (v,\psi ), \quad v \in H^1_0(\Gamma ) \end{aligned}$$

where $\psi =e = u -u_h^l$ on $\Gamma _h^l$ and $\psi =0$ on $\Gamma {\setminus } \Gamma _h^l$, and we extend $\phi$ using the extension operator to $U_{\delta _0}(\Gamma )\cap \widetilde{\Gamma }$. Then we have the stability estimate

3.144 $$\begin{aligned} \Vert \phi \Vert _{H^2(\Gamma \cup {\Gamma _h^l}{)}} \lesssim \Vert \phi \Vert _{H^2(\Gamma )} \lesssim \Vert \psi \Vert _{{\Gamma _h^l}} = \Vert e \Vert _{{\Gamma _h^l}} \end{aligned}$$

where the first inequality follows from the stability (3.29) of the extension of $\phi$ and the second is the elliptic regularity of the solution to the dual problem.

We obtain the following representation formula for the error

3.145 $$\begin{aligned} \Vert e\Vert ^2_{{\Gamma _h^l}}&= (e,\psi +\Delta \phi )_{{\Gamma _h^l}} - (e,\Delta \phi )_{{\Gamma _h^l}} \end{aligned}$$

3.146 $$\begin{aligned}&=(e,\psi +\Delta \phi )_{{\Gamma _h^l}{\setminus } \Gamma } + (\nabla e,\nabla \phi )_{{\Gamma _h^l}} - (e,\nu _{\partial {\Gamma _h^l}}\cdot \nabla \phi )_{\partial {\Gamma _h^l}} \end{aligned}$$

3.147 $$\begin{aligned}&=\underbrace{(e,\psi +\Delta \phi )_{{\Gamma _h^l}{\setminus } \Gamma }}_{I} + \underbrace{a_{{\Gamma _h^l}}(e,\phi )}_{II} + \underbrace{(\nu _{\partial {\Gamma _h^l}}\cdot \nabla _\Gamma e, \phi )_{\partial {\Gamma _h^l}} - \beta h^{-1} (e,\phi )_{\partial {\Gamma _h^l}}}_{III} \end{aligned}$$

$$\square $$

**Term **$\varvec{I}$ We have the estimates

3.148 $$\begin{aligned} |I|&=|(e,\psi +\Delta \phi )_{{\Gamma _h^l}{\setminus } \Gamma }| \end{aligned}$$

3.149 $$\begin{aligned}&\lesssim \Vert e\Vert _{{\Gamma _h^l}{\setminus } \Gamma } \Vert \psi +\Delta \phi \Vert _{{\Gamma _h^l}{\setminus } \Gamma } \end{aligned}$$

3.150 $$\begin{aligned}&\lesssim \Big (\delta _h^2 \Vert \nabla _\Gamma e \Vert ^2_{{\Gamma _h^l}{\setminus } \Gamma } + \delta _h \Vert e\Vert ^2_{\partial {\Gamma _h^l}} \Big )^{1/2} \Big (\Vert \psi \Vert _{{\Gamma _h^l}{\setminus } \Gamma } + \Vert \Delta \phi \Vert _{{\Gamma _h^l}{\setminus } \Gamma } \Big ) \end{aligned}$$

3.151 $$\begin{aligned}&\lesssim \Big ( (\delta _h^2 + h \delta _h) |||e |||_{\Gamma _h^l}^2 \Big )^{1/2} \Big ( \Vert e\Vert _{{\Gamma _h^l}{\setminus } \Gamma } + \Vert \phi \Vert _{H^2(\Gamma )} \Big ) \end{aligned}$$

3.152 $$\begin{aligned}&\lesssim \underbrace{(h^{-2} \delta _h + h^{-1}\delta _h)^{1/2}}_{\lesssim 1} h |||e |||_{\Gamma _h^l}\Vert e \Vert _{{\Gamma _h^l}} \end{aligned}$$

Here we used the Poincaré estimate (3.71) together with the definition of the energy norm to conclude that $\Vert e\Vert _{{\Gamma _h^l}{\setminus } \Gamma } \lesssim h |||e |||_{\Gamma _h^l}$, the stability (3.144) of the dual problem to conclude that $\Vert \psi + \Delta \phi \Vert _{{\Gamma _h^l}{\setminus } \Gamma } \lesssim \Vert e \Vert _{{\Gamma _h^l}}$, and finally the fact $\delta _h \lesssim h^{k+1}$.

**Term **$\varvec{I}\varvec{I}$ Adding and subtracting an interpolant we obtain

3.153 $$\begin{aligned} |II|&= \left| a_{\Gamma _h^l}\left( e,\phi - \pi _h^l \phi \right) + a_{\Gamma _h^l}\left( e,\pi _h^l \phi \right) \right| \end{aligned}$$

3.154 $$\begin{aligned}&\lesssim |||e |||_{\Gamma _h^l}|||\phi - \pi _h^l \phi |||_{\Gamma _h^l}+ \left| a_{\Gamma _h^l}\left( e,\pi _h^l \phi \right) \right| \end{aligned}$$

3.155 $$\begin{aligned}&\lesssim h |||e |||_{\Gamma _h^l}\Vert \phi \Vert _{H^2(\Gamma )} + \left| a_{\Gamma _h^l}\left( e,\pi _h^l \phi \right) \right| \end{aligned}$$

3.156 $$\begin{aligned}&\lesssim h |||e |||_{\Gamma _h^l}\Vert e\Vert _{{\Gamma _h^l}} + \left| a_{\Gamma _h^l}\left( e,\pi _h^l \phi \right) \right| \end{aligned}$$

For the second term on the right hand side we first note that using Lemma 3.5 and Lemma 3.7 we have the estimates

3.157 $$\begin{aligned} a_{\Gamma _h^l}\left( e,\pi _h^l\phi \right)&= a_{\Gamma _h^l}\left( u,\pi _h^l \phi \right) - a_{\Gamma _h^l}\left( u_h^l,\pi _h^l \phi \right) \end{aligned}$$

3.158 $$\begin{aligned}&= a_{\Gamma _h^l}\left( u,\pi _h^l \phi \right) - l_{\Gamma _h^l}\left( \pi _h^l \phi \right) \nonumber \\&\quad + l_{\Gamma _h^l}\left( \pi _h^l \phi \right) - l_{\Gamma _h}\left( \pi _h \phi \right) \nonumber \\&\quad + a_{\Gamma _h}\left( u_h,\pi _h \phi \right) - a_{\Gamma _h^l}\left( u_h^l,\pi _h^l \phi \right) \end{aligned}$$

3.159 $$\begin{aligned}&\lesssim \delta _h \Vert u \Vert _{H^{k+1}(\Gamma )} \underbrace{\Big (\Vert \nabla _\Gamma \pi _h^l \phi \Vert _{{\Gamma _h^l}} + h^{-1/2} |||\pi _h^l \phi |||_{\partial {\Gamma _h^l}} \Big )}_{II_1} \nonumber \\&\quad + h^{k+1} \Big ( \Vert f \Vert _{\Gamma } + \Vert g \Vert _{\partial \Gamma } \Big ) \underbrace{\Big (\Vert {\nabla _{\Gamma _h}} \pi _h \phi \Vert _{{{\Gamma _h}}} + h^{-1/2} |||\pi _h \phi |||_{{\partial {\Gamma _h}}} \Big )}_{II_2} \nonumber \\&\quad + h^{k+1} \underbrace{\Big (\Vert \nabla _{\Gamma _h}u_h \Vert _{\Gamma _h}+ h^{1/2}|||u_h |||_{\partial {\Gamma _h}} \Big )}_{II_3} \underbrace{\Big ( \Vert \nabla _{\Gamma _h}\pi _h \phi \Vert _{\Gamma _h}+ h^{-1/2} |||\pi _h \phi |||_{\partial {\Gamma _h}} \Big )}_{II_2} \end{aligned}$$

3.160 $$\begin{aligned}&\lesssim (\delta _h + h^{k+1}) \Vert u \Vert _{H^{k+1}(\Gamma )} \Vert e\Vert _{{\Gamma _h^l}} + h^{k+1} \Big ( \Vert f \Vert _{\Gamma } + \Vert g \Vert _{\partial \Gamma } \Big ) \Vert e\Vert _{{\Gamma _h^l}} \end{aligned}$$

where we finally used the estimates

3.161 $$\begin{aligned} II_1 \sim II_2 \lesssim \Vert e \Vert _{{\Gamma _h^l}}, \quad II_3 \lesssim \Vert u \Vert _{H^{k+1}(\Gamma )} \end{aligned}$$

In order to verify the estimates of Terms $II_1-II_3$, we first prove the trace inequality

3.162 $$\begin{aligned} \Vert v \Vert _{\partial {\Gamma _h^l}} \lesssim \Vert v \Vert _{H^1(\Gamma )} \quad v \in H^1(\Gamma ) \end{aligned}$$

where the hidden constant is independent of $h \in (0,h_0]$, for $h_0$ small enough. Adding and subtracting $v \circ p_{\partial \Gamma }$, using the triangle inequality, we obtain

3.163 $$\begin{aligned} \Vert v \Vert ^2_{\partial {\Gamma _h^l}}&\lesssim \Vert v - v \circ p_{\partial \Gamma }\Vert ^2_{\partial {\Gamma _h^l}} + \Vert v \circ p_{\partial \Gamma }\Vert ^2_{\partial {\Gamma _h^l}} \end{aligned}$$

3.164 $$\begin{aligned}&\lesssim \delta _h \Vert v \Vert ^2_{H^1\big (U_{\delta _h}^l\big (\partial \Gamma \big ) \cap \widetilde{\Gamma }\big )} + \Vert v \Vert ^2_{\partial \Gamma } \end{aligned}$$

3.165 $$\begin{aligned}&\lesssim \delta _h \Vert v \Vert ^2_{H^1\big (U_{\delta _h}^l\big (\partial \Gamma \big ) \cap \widetilde{\Gamma }\big )} + \Vert v \Vert ^2_{H^1\big (\Gamma \big )} \end{aligned}$$

3.166 $$\begin{aligned}&\lesssim \Vert v \Vert ^2_{H^1\big (U_{\delta _h}^l\big (\partial \Gamma \big ) \cap \widetilde{\Gamma }\big ) \cup \Gamma } \end{aligned}$$

3.167 $$\begin{aligned}&\lesssim \Vert v \Vert ^2_{H^1\big (\Gamma \big )} \end{aligned}$$

where in (3.164) we used equivalence of norms (3.18) for the second term and for the first term we used estimate (3.62), in (3.165) we used the trace inequality $$\Vert v \Vert _{\partial \Gamma } \lesssim \Vert v \Vert _{H^1(\Gamma )},$$$$v \in H^1(\Gamma )$$, for the second term, in (3.166) we used the bound $$\delta _h\lesssim h^{k+1} \lesssim h_0^{k+1} \lesssim 1$$ collected the two contributions in one norm, and in (3.167) we used the stability (3.29) of the extension of *v*. This concludes the proof of (3.162).

**Terms**$\varvec{I}\varvec{I}_{\varvec{1}}$**and**$\varvec{I}\varvec{I}_{\varvec{2}}$ Using equivalence of norms

3.168 $$\begin{aligned} II_1 = \Vert \nabla _{\Gamma _h}\pi _h \phi \Vert _{{{\Gamma _h}}} + h^{-1/2} |||\pi _h \phi |||_{\partial {\Gamma _h}} \sim \Vert \nabla _\Gamma \pi _h^l \phi \Vert _{\Gamma _h^l}+ h^{-1/2}|||\pi _h^l \phi |||_{\partial {\Gamma _h^l}} = II_2 \end{aligned}$$

The first term on the right hand side is handled as in (3.174)–(3.177) and the second is bounded as follows

3.169 $$\begin{aligned} h^{-1}|||\pi _h^l \phi |||^2_{\partial {\Gamma _h^l}}&\lesssim h^{-1}|||\pi _h^l \phi - \phi |||^2_{\partial {\Gamma _h^l}} + h^{-1}|||\phi |||^2_{\partial {\Gamma _h^l}} \end{aligned}$$

3.170 $$\begin{aligned}&\lesssim h \Vert \phi \Vert ^2_{H^2\left( {\Gamma _h^l}\right) } + \Vert \nabla _\Gamma \phi \Vert ^2_{\partial {\Gamma _h^l}} + h^{-2} \Vert \phi \Vert ^2_{\partial {\Gamma _h^l}} \end{aligned}$$

3.171 $$\begin{aligned}&\lesssim h \Vert \phi \Vert ^2_{H^2\left( {\Gamma _h^l}\right) } + \Vert \phi \Vert ^2_{H^2(\Gamma )} + h^{-2}\delta _h^2 \Vert \phi \Vert ^2_{H^2(\Gamma )} \end{aligned}$$

3.172 $$\begin{aligned}&\lesssim \underbrace{(h + 1 + h^{-2}\delta _h^2)}_{\lesssim 1} \Vert \phi \Vert ^2_{H^2(\Gamma )} \end{aligned}$$

where we added and subtracted the exact solution, used the interpolation error estimate (3.34) for the first term on the right hand side, the trace inequality (3.162) for the second term, the fact that $\phi =0$ on $\Gamma$ together with (3.52) for the third term, and finally stability of the extension operator (3.29). Thus we conclude that

3.173 $$\begin{aligned} \Vert \nabla _\Gamma \pi _h^l \phi \Vert _{\Gamma _h^l}+ h^{-1/2}|||\pi _h^l \phi |||_{\partial {\Gamma _h^l}} \lesssim \Vert \phi \Vert _{H^2(\Gamma )} \lesssim \Vert e\Vert _{{\Gamma _h^l}} \end{aligned}$$

**Term**$\varvec{I}\varvec{I}_{\varvec{3}}$ We have

3.174 $$\begin{aligned} \Vert \nabla _{\Gamma _h}u_h \Vert _{\Gamma _h}+ h^{1/2}|||u_h |||_{\partial {\Gamma _h}}&\sim \Vert \nabla _\Gamma u_h^l \Vert _{\Gamma _h^l}+ h^{1/2}|||u_h^l |||_{\partial {\Gamma _h^l}} \end{aligned}$$

3.175 $$\begin{aligned}&\le \Vert \nabla _\Gamma (u_h^l - u) \Vert _{\Gamma _h^l}+ h^{1/2}|||(u_h^l - u) |||_{\partial {\Gamma _h^l}} \nonumber \\&\quad + \Vert \nabla _\Gamma u \Vert _{\Gamma _h^l}+ h^{1/2}|||u |||_{\partial {\Gamma _h^l}} \end{aligned}$$

3.176 $$\begin{aligned}&\lesssim h^k \Vert u \Vert _{H^{k+1}(\Gamma )} + \Vert u \Vert _{H^2(\Gamma )} \end{aligned}$$

3.177 $$\begin{aligned}&\lesssim \Vert u \Vert _{H^{k+1}(\Gamma )} \end{aligned}$$

where we used equivalence of norms, added and subtracted the exact solution, used the triangle inequality and the energy norm error estimate (3.137), and the estimate

3.178 $$\begin{aligned} h |||u |||^2_{\partial {\Gamma _h^l}}&= h^2 \Vert \nabla _\Gamma u \Vert ^2_{\partial {\Gamma _h^l}} + \Vert u \Vert ^2_{\partial {\Gamma _h^l}} \end{aligned}$$

3.179 $$\begin{aligned}&\lesssim h^2 \Vert \nabla _\Gamma u \Vert ^2_{H^1(\Gamma )} + \Vert u \Vert ^2_{H^1(\Gamma )} \end{aligned}$$

3.180 $$\begin{aligned}&\lesssim \Vert u \Vert ^2_{H^2(\Gamma )} \end{aligned}$$

where we used (3.162).

**Term **$\varvec{I}\varvec{I}\varvec{I}$ Using the Cauchy–Schwarz inequality we get

3.181 $$\begin{aligned} |III|&\le h|||e |||_{\Gamma _h^l}h^{-3/2}\Vert \phi \Vert _{\partial {\Gamma _h^l}} \lesssim h|||e |||_{\Gamma _h^l}h^{-3/2}\delta _h \Vert \phi \Vert _{H^2(\Gamma )} \lesssim h|||e |||_{\Gamma _h^l}\Vert e \Vert _{{\Gamma _h}} \end{aligned}$$

#### Remark 3.8

Our results directly extends to the case of a Neumann or Robin condition

3.182 $$\begin{aligned} \nu \cdot \nabla _\Gamma u = g_N - \kappa u \end{aligned}$$

where $\kappa \ge 0$ on a part of the boundary. Essentially we need to modify the quadrature term estimates to account for the terms involved in the weak statement of the Robin condition. These terms are very similar to the terms involved in the Nitsche formulation for the Dirichlet problem and may be estimated in the same way.

#### Remark 3.9

Strong implementations of the Dirichlet boundary condition may also be considered in our framework. In this remark we summarize the main modifications in the formulation of the method and in the analysis. To formulate a finite element method with strong Dirichlet boundary conditions we need to interpolate the Dirichlet data and construct a suitable interpolation operator. Then we formulate the method and finally we discuss the modifications in the theoretical results.

**Interpolation** We recall that in the construction of the Scott–Zhang interpolation operator, see [22], to each Lagrange node $x_i$ we associate a simplex $S_i$, such that $x_i \in S_i$ and $S_i$ is a triangle for nodes $x_i \in \Gamma _h {\setminus } \partial {\Gamma _h}$ in the interior of the discrete domain and $S_i$ is an edge on the boundary $\partial {\Gamma _h}$ when $x_i \in \partial {\Gamma _h}$. Let $\{\varphi _{i,k}\}$ be the Lagrange basis associated with the simplex $S_i$ and let $\{\psi _{i,l}\}$ be the dual basis such that $(\varphi _k,\psi _l)_{S_i} = \delta _{kl}$. We let $\psi _i$ denote the dual basis function $\psi _{i,l}$ associated with node *i*, then the nodal value $v(x_i) = ( v, \psi _{i} )_{S_i}$, for $v \in V_h$. The interpolation operator is defined by

3.183 $$\begin{aligned} I_h u = \sum _{i=1}^N I_h u (x_i) \varphi _i \end{aligned}$$

where *N* is the number of nodes and the nodal values are defined by

3.184 $$\begin{aligned} I_h u (x_i) = {\left\{ \begin{array}{ll} (u \circ p (x_i),\psi _{i})_{S_i} &{} x_i \in {\Gamma _h}{\setminus } \partial {\Gamma _h}\\ (g \circ p_{\partial \Gamma } ( x_i ), \psi _{i})_{S_i} &{} x_i \in \partial {\Gamma _h}\end{array}\right. } \end{aligned}$$

where we note that we use the closest point mapping $p_{\partial \Gamma }$ for the nodes on the boundary and *p* for the nodes in the interior. We have the interpolation error estimate

3.185 $$\begin{aligned} \left\| u - (I_h u)^l \right\| _{H^m\left( {\Gamma _h^l}\right) } \lesssim h^{k+1-m} \Vert u \Vert _{H^{k+1}(\Gamma )}, \quad m = 0,1 \end{aligned}$$

To verify that (3.185) holds we note that it follows from (3.32) and the stability of the extension operator (3.29), that the Scott–Zhang interpolation operator ${\pi }_h$, defined in (3.30), satisfies the estimate

3.186 $$\begin{aligned} \left\| u - ({\pi }_hu)^l \right\| _{H^m\left( {\Gamma _h^l}\right) } \lesssim h^{k+1-m} \Vert u \Vert _{H^{k+1}(\Gamma )} \end{aligned}$$

for $$m=0,1$$.

We next note that $I_h u (x_i)- {\pi }_hu (x_i) = 0$ for $x_i \in {\Gamma _h}{\setminus } \partial {\Gamma _h}$, see the definition of the nodal values (3.31) and (3.184), and we have the inverse estimate

3.187 $$\begin{aligned} \Vert I_h u - {\pi }_hu \Vert ^2_{\Gamma _h}&\lesssim h \Vert I_h u - {\pi }_hu \Vert ^2_{\partial \Gamma _h} \end{aligned}$$

since for any element *K* with at least one vertex at the boundary $\partial {\Gamma _h}$ we have the inverse estimates

3.188 $$\begin{aligned} \Vert v \Vert ^2_{K} \lesssim h^2 \Vert v \Vert ^2_{L^\infty (K)} \lesssim h^2 \max _{x_i \in \partial K \cap \partial {\Gamma _h}} | v(x_i) |^2 \lesssim h \Vert v \Vert ^2_{S_i} \end{aligned}$$

To estimate the boundary term on the right hand side in (3.187) we proceed as follows

3.189 $$\begin{aligned} \Vert I_h u - {\pi }_hu \Vert ^2_{\partial {\Gamma _h}}&\lesssim \sum _{i=1}^{N_b} h | (u \circ p, \psi _{i})_{S_i} - (u \circ p_{\partial \Gamma },\psi _i)_{S_i} |^2 \end{aligned}$$

3.190 $$\begin{aligned}&\lesssim \sum _{i=1}^{N_b} h | (u - u \circ \widetilde{p}_{\partial \Gamma },\psi _i^l)_{S_i^l} |^2 \end{aligned}$$

3.191 $$\begin{aligned}&\lesssim \sum _{i=1}^{N_b} \Vert u - u \circ \widetilde{p}_{\partial \Gamma }\Vert _{S_i^l} \underbrace{h \Vert \psi _i \Vert ^2_{S_i}}_{\lesssim 1} \end{aligned}$$

3.192 $$\begin{aligned}&\lesssim \Vert u - u \circ \widetilde{p}_{\partial \Gamma }\Vert ^2_{\Gamma _h^l} \end{aligned}$$

3.193 $$\begin{aligned}&\lesssim h^{2(k+1)} \Vert u \Vert ^2_{H^2(\Gamma )} \end{aligned}$$

where we estimated the $L^2$ norm in terms of the nodal values on the boundary with $N_b$ the number of nodes at the boundary, expressed the difference using $\widetilde{p}_{\partial \Gamma }$, used the Cauchy–Schwarz inequality and the bound $\Vert \psi _i \Vert ^2_{S_i} \lesssim h^{-1}$, used the fact that each simplex (edge) $S_i$ occur in the sum a bounded number of times, and finally we used Lemma 3.3. We thus conclude that

3.194 $$\begin{aligned} \Vert I_h u - {\pi }_hu \Vert ^2_{\Gamma _h} \lesssim h^{2k+3} \Vert u \Vert ^2_{H^2(\Gamma )} \lesssim h^{2k+2} \Vert u \Vert ^2_{H^2(\Gamma )} \end{aligned}$$

where we used the fact $h \in (0,h_0]$. Adding and subtracting ${\pi }_hu$, using the triangle inequality, followed by the interpolation estimate (3.186) and the estimate of the difference between the interpolants (3.194) we finally obtain

3.195 $$\begin{aligned} \big \Vert u^e - I_h u^e \big \Vert ^2_{H^m({\Gamma _h})}&\lesssim \big \Vert u^e - {\pi }_hu^e \big \Vert ^2_{H^m({\Gamma _h})} + \big \Vert I_h u^e - {\pi }_hu^e \big \Vert ^2_{H^m({\Gamma _h})} \end{aligned}$$

3.196 $$\begin{aligned}&\lesssim \big \Vert u^e - {\pi }_hu^e \big \Vert ^2_{H^m({\Gamma _h})} + h^{-2m} \big \Vert I_h u^e - {\pi }_hu^e \big \Vert ^2_{L^2({\Gamma _h})} \end{aligned}$$

3.197 $$\begin{aligned}&\lesssim h^{2(k+1-m)} \big \Vert u \big \Vert ^2_{H^{k+1}(\Gamma )} + h^{2(k+1-m)} \big \Vert u \big \Vert ^2_{H^{2}(\Gamma )} \end{aligned}$$

3.198 $$\begin{aligned}&\lesssim h^{2(k+1-m)} \big \Vert u \big \Vert ^2_{H^{k+1}(\Gamma )} \end{aligned}$$

**Method** To formulate the method we define the discrete Dirichlet data

3.199 $$\begin{aligned} g_h = I_h (g \circ p_{\partial \Gamma }) \end{aligned}$$

Introducing the trial and test spaces

3.200 $$\begin{aligned} V_{h,g_h} = \{ v \in V_h {:}\; v|_{\partial {\Gamma _h}} = g_h\}, \quad V_{h,0} = \{ v \in V_h {:}\; v|_{\partial {\Gamma _h}} = 0\} \end{aligned}$$

we have the finite element method: find $u_h \in V_{h,g_h}$ such that

3.201 $$\begin{aligned} a_{{\Gamma _h}}(u_h,v) = l_{{\Gamma _h}}(v) \quad \forall v \in V_{h,0} \end{aligned}$$

where

3.202 $$\begin{aligned} a_{\Gamma _h}(v,w) = (\nabla _{\Gamma _h}v,\nabla _{\Gamma _h}w)_{\Gamma _h}, \quad l_{\Gamma _h} (w) = (f^e,w)_{\Gamma _h} \end{aligned}$$

**Error estimates** In the analysis the following modifications are done:

- The energy norms are defined by
  3.203 $$\begin{aligned} |||v |||_{{\Gamma _h}} = \Vert \nabla _{\Gamma _h}v \Vert _{{\Gamma _h}}, \quad |||v |||_{{\Gamma _h^l}} = \Vert \nabla _\Gamma v \Vert _{{\Gamma _h^l}} \end{aligned}$$
- In the Strang Lemma the lifted forms are simplified to
  3.204 $$\begin{aligned} a_{\Gamma _h^l}(v,w) = (\nabla _\Gamma v,\nabla _\Gamma w)_{\Gamma _h^l}, \quad l_{\Gamma _h^l} (w) = (f,w)_{\Gamma _h^l} \end{aligned}$$
  and observing that the discrete error $I_h u - u_h \in V_{h,0}$ we have
  3.205 $$\begin{aligned} |||I_h u - u_h |||_{\Gamma _h} \lesssim \sup _{v \in V_{h,0} {\setminus } \{0\}} \frac{a_{{\Gamma _h}}(I_h u - u_h,v)}{|||v |||_{{\Gamma _h}}} \end{aligned}$$
  and thus we may derive the Strang Lemma in the same way as for the Nitsche condition.
- For the consistency error we have the simplified expression
  3.206 $$\begin{aligned} a_{\Gamma _h^l}(u,v^l) - l_{\Gamma _h^l}(v^l) = -(f+\Delta _\Gamma u ,v^l)_{\Gamma _h^l {\setminus } \Gamma } \end{aligned}$$
  and the estimate
  3.207 $$\begin{aligned} \Big | a_{\Gamma _h^l}(u,v^l) - l_{\Gamma _h^l}(v^l) \Big | \lesssim \delta _h \Vert u \Vert _{H^2(\Gamma )} |||v |||_{{\Gamma _h}} \end{aligned}$$
- The quadrature estimates in Lemma 3.7) are simplified to
  3.208 $$\begin{aligned} \Big | a_{{\Gamma _h^l}}(v^l,w^l) - a_{{\Gamma _h}}(v,w) \Big |&\lesssim h^{k+1} |||v |||_{{\Gamma _h}} |||w |||_{{\Gamma _h}} \quad \forall v, w \in V_h \end{aligned}$$
  3.209 $$\begin{aligned} \Big | l_{{\Gamma _h^l}}(v^l) - l_{{\Gamma _h}}(v)\Big | \lesssim h^{k+1} \Big ( \Vert f\Vert _\Gamma + \Vert g\Vert _{\partial \Gamma } \Big ) |||v |||_{\Gamma _h}\quad \forall v \in V_h \end{aligned}$$

Combining these results we obtain energy and $L^2$ error estimates of the same form as in Theorems 3.1 and 3.2.

#### Remark 3.10

It is not necessary to use the same order of polynomials in the mappings of the elements and the finite element space. We may instead use polynomials of order $k_g$ for the geometry approximation and $k_u$ for the finite element space. See [15] for an example of an application where different approximations order are used in the context of the Darcy problem on a closed surface. Essentially, this affects the consistency error estimate in Lemma 3.5, where we will have $\delta _h \sim h^{k_g+1}$, and the quadrature error estimates in Lemma 3.7, where we will replace *k* by $k_g$. Clearly to obtain optimal order convergence in both the energy and the $L^2$ norm we must use the same or higher order polynomials in the mappings as in the finite element space, i.e., $k_g\ge k_u$.

## Numerical examples

**Model problem** We consider the Laplace–Beltrami problem on a torus with a part removed. To express points on the torus surface we use toroidal coordinates $\{\theta ,\phi \}$ defined such that the corresponding Cartesian coordinates are given by

4.1 $$\begin{aligned} x_1= (R+r \cos (\theta )) \cos (\phi ), \quad x_2 = (R+r \cos (\theta )) \sin (\phi ), \quad x_3 = r \sin (\theta ) \end{aligned}$$

with constants $R=1$ and $r=0.4$. The boundary $\partial \Gamma$ is defined by the curves

4.2 $$\begin{aligned} \phi _1(\theta ) = 0.2 \cos (N_1 \theta ) \quad \text {and} \quad \phi _2(\theta ) = 0.2 \cos (N_2 \theta ) + 0.6 (2R\pi ) \end{aligned}$$

where we choose $N_1=4$ and $N_2=3$. In turn the domain $\Gamma$ is given by

4.3 $$\begin{aligned} \Gamma = \left\{ \theta ,\phi {:}\; 0 \le \theta < 2\pi \, , \ \phi _1 \le \phi \le \phi _2 \right\} \end{aligned}$$

We manufacture a problem with a known analytic solution by prescribing the solution

4.4 $$\begin{aligned} u = \cos (3\phi +5\theta )\sin (2\theta ) \end{aligned}$$

and compute the corresponding load *f* by using the identity $f=-\Delta _\Gamma u$. The Dirichlet boundary data on $\partial \Gamma$ is directly given by $g=u|_{\partial \Gamma }$. Note that (4.4) is smooth and defined on the complete torus so clearly the stability estimates (2.17) and (3.29) for *f* and *u* both hold.

**Fig. 1:**
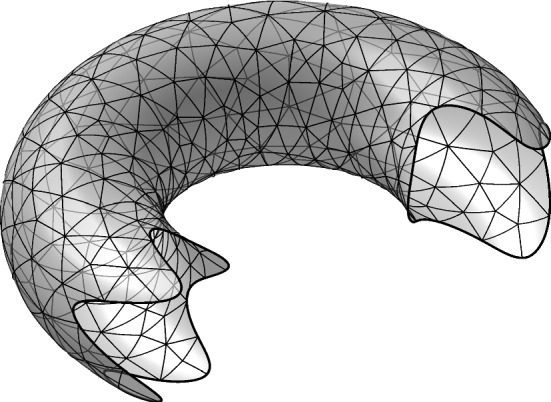
Mesh for the model problem using geometric interpolation order $k=3$ and meshsize $h=1/4$

**Geometry discretization**$\varvec{\Gamma }_{\varvec{h}}$ We construct higher order ($k>1$) geometry approximations ${\Gamma _h}$ from an initial piecewise linear mesh ($k=1$) by adding nodes for higher order Lagrange interpolation through linear interpolation between the facet vertices. All mesh nodes are moved to the exact surface by the closest point map *p*(*x*) and then the boundary is corrected such that the nodes on the discrete boundary $\partial \Gamma _h$ coincide with the exact boundary $\partial \Gamma$. A naive approach for the correction is to just move nodes on the boundary of the mesh to the exact boundary. For our model problem we let the corrected boundary nodal points be given by the toroidal coordinates $\{\theta ,\phi _i(\theta )\}$. This may however give isoparametric mappings with bad quality or negative Jacobians in some elements, especially in coarser meshes and higher order interpolations where the element must be significantly deformed to match the boundary. We therefore use a slightly more refined procedure where interior nodes are positioned inside the element according to a quadratic map aligned to the boundary, rather than using linear interpolation over the facet. In Fig. 1 a coarse mesh for the model problem using $k=3$ interpolation is presented.

**Fig. 2:**
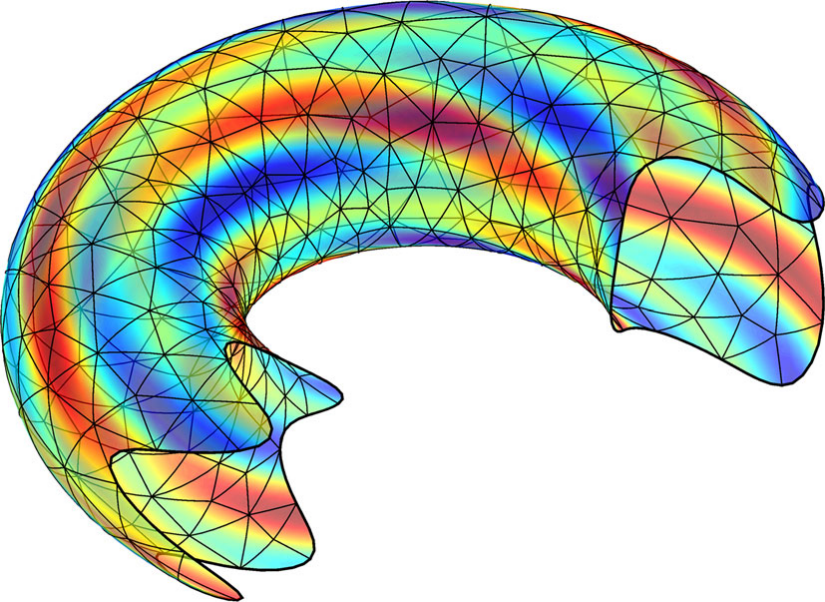
Numerical solution for the model problem using $k=3$ and meshsize $h=1/4$

**Numerical study** The numerical solution for the model problem with $k=3$ and $h=1/4$ is visualized in Fig. 2. We choose the Nitsche penalty parameter $\beta =10^4$. This large value was chosen in order to achieve the same size of the error as when strongly enforcing the Dirichlet boundary conditions and using $k=4$.

**Fig. 3:**
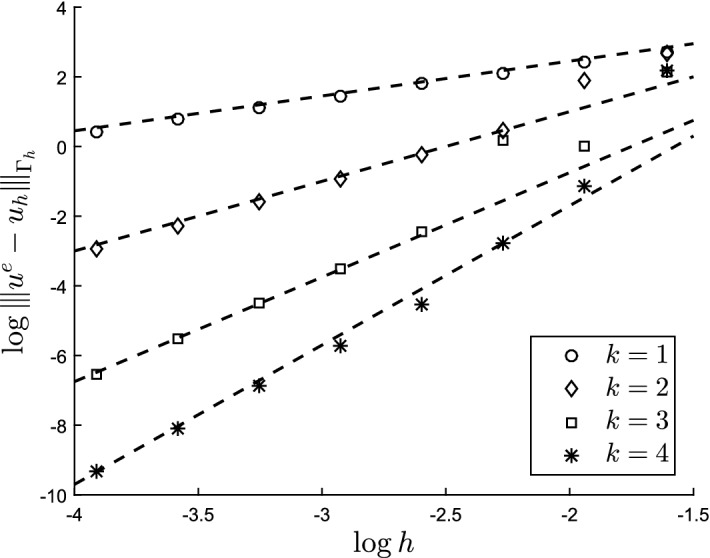
Convergence study of the model problem in energy norm with reference lines proportional to $h^{k}$. Note the instability in convergence rate for coarse meshes and higher order geometry approximation

**Fig. 4:**
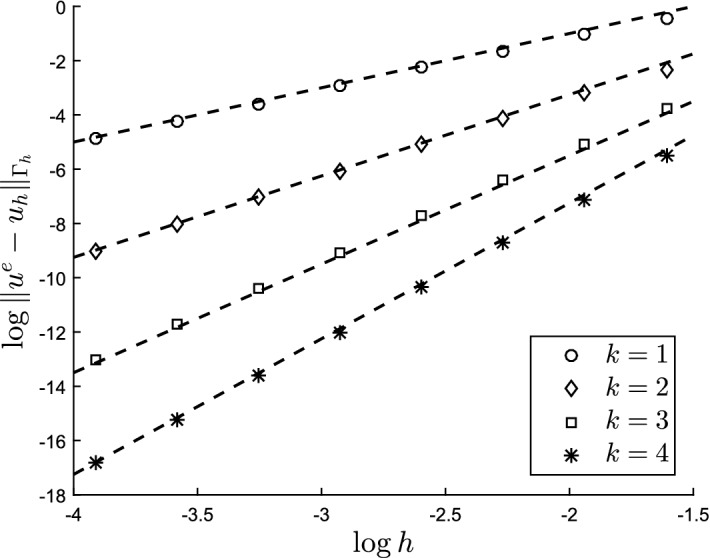
Convergence study of the model problem in $L^2$ norm with reference lines proportional to $h^{k+1}$

The results for the convergence studies in energy norm and $L^2$ norm are presented in Figs. 3 and 4. These indicate convergence rates of $O(h^k)$ in energy norm and $O(h^{k+1})$ in $L^2$ norm which by norm equivalence is in agreement with Theorem 3.1 and Theorem 3.2, respectively. On coarse meshes we note some inconsistencies in the energy norm results when using higher order interpolations. We attribute this effect to large derivatives of the mappings used to make the element fit the boundary which may arise in some elements for coarse meshes that are large in comparison to the variation of the boundary. When the boundary is better resolved we retain the proper convergence rates. Note also that the Jacobian of the mapping is involved in the computation of the gradient which explains that we see this behavior in the energy norm but not in the $L^2$ norm.

**Fig. 5:**
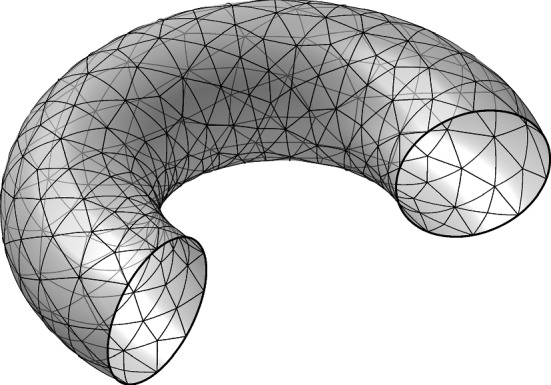
Mesh for a simplified version of the model problem ($N_1=N_2=0$) using geometric interpolation order $k=3$ and meshsize $h=1/4$. For this problem ${\Gamma _h^l}=\Gamma$

**Fig. 6:**
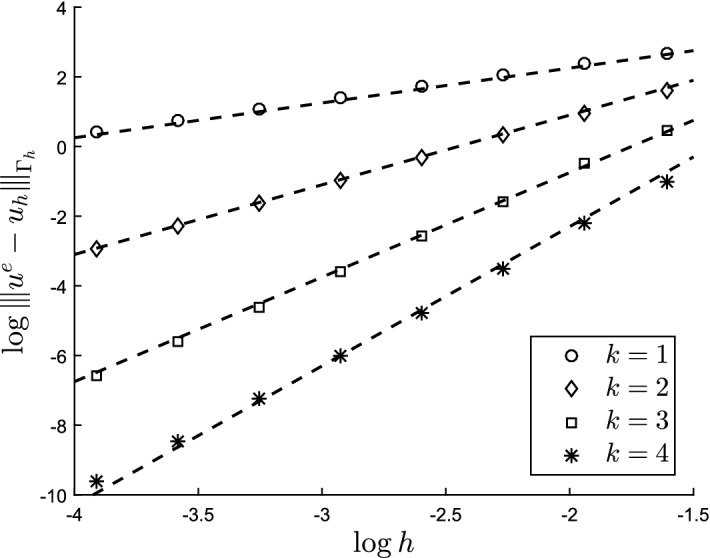
Convergence study for a simplified version of the model problem ($N_1=N_2=0$) in energy norm with reference lines proportional to $h^{k}$. Note that there is no instability in convergence rate for coarse meshes

In the special case ${\Gamma _h^l}= \Gamma$, such as the simplified model problem, obtained by taking parameters $N_1=N_2=0$ in the boundary description (4.2), illustrated by the mesh in Fig. 5, no correction of boundary nodes onto $\partial \Gamma$ is needed. In that case the energy error aligns perfectly with the reference lines also for coarse meshes and higher order geometry approximations, see Fig. 6.
